# Leakage current characteristics in estimating insulator reliability: experimental investigation and analysis

**DOI:** 10.1038/s41598-022-17792-x

**Published:** 2022-09-02

**Authors:** Ali Ahmed Salem, Kwan Yiew Lau, Wan Rahiman, Zulkurnain Abdul-Malek, Samir A. Al-Gailani, R. Abd Rahman, Salem Al-Ameri

**Affiliations:** 1 Institute of High Voltage and High Current, School of Electrical Engineering, Universiti Teknologi Malaysia, 81310 Johor Bahru, Malaysia; 2 School of Electrical and Electronic Engineering, Universiti Sains Malaysia, 14300 Nibong Tebal, Penang Malaysia; 3 Faculty of Electrical and Electronic Engineering, University Tun Hussein Onn Malaysia, 86400 Batu Pahat, Malaysia

**Keywords:** Engineering, Electrical and electronic engineering

## Abstract

The monitoring of leakage current (LC) and voltage characteristics in transmission line insulators is regarded as a good technique for anticipating the physical state of in-service insulators. In the current work, the temporal and frequency characteristics of LC and voltage under various situations were derived for assessing the health condition of porcelain, glass, and silicone rubber insulators. The contamination severity indicated by soluble deposit density, wetting level (Wt), non-soluble deposit density, and uneven pollution distribution (P_u_/P_L_) were chosen as the environmental factors that impact the insulators. Six criteria were utilized to evaluate the physical state of the insulators, with four of those derived from the LC signal in the time domain, namely, the LC signal peak (C_1_), the phase shift between applied voltage and LC (C_2_), the LC signal slope between two consecutive peaks (C_3_), and the crest factor (C_4_). The remaining two indices, namely, the total harmonics distribution (C_5_) and the harmonics ratio indicator (C_6_), were obtained from the frequency domain of the LC signal. In addition, the flashover voltage index (C_7_) was also employed. The LC indicators were then classified based on the laboratory test results to reflect the physical state of the insulators. The findings revealed that the proposed indicators had an important impact in determining the physical state of the insulators. Furthermore, a confusion matrix was created for the test and prediction data using the suggested indicators to determine the effectiveness of each indicator.

## Introduction

One of the important parts of electrical power transmission systems is the transmission line insulators. The efficiency of the transmission systems is related to the health of the insulation systems. Accordingly, it is important to observe different environmental factors, including the type of materials, dampness, and pollutants, that have a considerable influence on the efficiency and health of the high voltage transmission lines insulators that can directly affect the performance of the transmission systems. For example, pollution deposition on the surface of the insulators results in the insulators being subject to a leakage current (LC) flux which leads to extensive discharge activity on the insulators' surface^1–6^. As a result, these discharge activities can escalate and generate a flashover occurrence, which might cause an electrical grid outage or even breakdown^7–9^. Therefore, it is necessary to monitor and assess the health condition of insulators in order to ensure the integrity of the insulator and the transmission system as a whole^10,11^. This improves the grid's performance and reduces the likelihood of a malfunction. The scientific community considers the study of the qualities of outdoor insulators and their long-term efficiency to be a relevant topic^12–15^.

There are several advantages of using the LC characteristics as a monitoring strategy. It considers different environmental parameters like surrounding temperature, humidity, pollution, and rainfall^16^. Furthermore, LC may be easily monitored online on an ongoing basis. Measuring and analyzing LC has stimulated the interest of many scientists as one of the online tests done on polluted insulators. In addition, the monitoring of LC was performed using a microwave reflectometer system for dry insulator surfaces^17^. This form of the monitoring system is however, expensive, making it impractical for power grids. Fontana et al*.*^18^ also introduced a technique that employed an antenna in LC monitoring on contaminated insulators by capturing the partial discharge electromagnetic radiation. The advantage of this technique is that, unlike other approaches, the parts utilized in it are not destroyed by flashover voltage. Furthermore, some studies in the literature presented different numerical indices for identifying the health condition of insulators, albeit that none of them examined the efficacy of such indicators on high voltage transmission line insulators. Only acoustic and thermal-based diagnostic methods, such as infrared thermal imaging, ultrasonic wave^19^, visible light images^20^, wireless-based system^21^, acoustic fault diagnosis^22^, network sensors^23^, and long-term analysis of LC using optical sensor^24^, have been used to assess the health of the overhead line insulators.

Moreover, the monitoring of insulator LC under contaminated conditions indicates the capacity to provide a reliable indicator between LC and the condition of high voltage insulators during service. In this regard, many studies have provided various ways of evaluating the physical state of insulators using a particular indication^16,25–27^. Recently, Palangar^28^ suggested the use of a combination of two LC harmonic ratios and phase angles to monitor the status of insulators. Although the results of the studies^28^ provide valuable ways for detecting the status of various types of insulators, estimation of the flashover probability, particularly for composite insulators, is not considered. Meanwhile, Zhao et al.^27^ employed the LC parameters such as the mean, peak, and standard deviation values to monitor the insulators' state. However, these characteristics were solely employed to calculate the size and density of the contaminated layer over the insulator surface. Another study^25^ discussed the contaminated insulator’s state by measuring the phase angle between the signals of LC and supplied voltage. According to the findings from the literature^26^, shift angle fluctuation is a good index for evaluating pollution and humidity differences on insulators' surfaces. Other relevant methodologies for predicting insulator pollution incidence include LC components such as the 3rd and 5th odd harmonics, and the total harmonic distortion (THD), which have been used by many researchers^29–31^. In this approach, the LC signal is analyzed in the frequency domain using the fast Fourier transform (FFT) and wavelet transform. The findings suggest that pollution causes the first and third frequency components, as well as THD^32^, to grow. In other words, increasing the LC harmonics raises THD, which changes according to the rate of contamination and harmonics of the applied voltage^33^.

Having an indicator that reflects the conditions of the insulators^16,20^ is critical. The use of the LC components in frequency and time domains to compute the necessary indicators were provided in the literature^34^. When calculating flashover occurrences, the indicator 5th/3rd and THD were used. In the case of silicone rubber (SIR) and glass insulators, the extent of pollution and the value of this indicator were shown to be highly correlated. However, disregarding the impacts of the 7th and 9th harmonics components might influence the accuracy of the results that represent the insulator state. Furthermore, a review of the literature revealed that no attempt has been made to analyze the conditions of insulators using index values that take into account the time waveform slope and harmonics up to the 9th component for LC, statistical analysis of the limitations of the indicator as well as a comparison of the performance of these indicators. Therefore, the goal of this research is to create indices based on the characteristics of LC and voltage while considering the major impact of environmental conditions on the performance of three different insulator types, namely porcelain, glass, and SIR. The pollution severity in terms of soluble deposit density (SDD), wetting level (Wt), non-soluble deposit density (NSDD), and non-uniform distribution contamination (P_u_/P_L_) were taken into consideration as the environmental factors that affect insulators during their service. The indices of LC generated by the impact of these environmental elements were compared. The suggested indicators were evaluated using the confusion matrix for the experimental data and prediction outcomes under 11 kV AC voltage. To sum up, the main contributions of this paper are as follows:

- Perform experimental tests to measure LC and flashover voltage of porcelain, glass, and SIR insulators under different levels of SDD, NSDD, Wt, and P_u_/P_L_.
- Extracting the characteristics of LC such as peak, harmonics, phase shift angle, slope, and THD.
- Extracting the characteristics of applied voltage such as flashover voltage and withstand voltage.
- Proposing six different indicators based on the characteristics of LC.
- Proposing indicator based on the characteristics of insulator voltage.
- Evaluating the insulator condition using the proposed indicators.
- Statistical evaluation using the confusion matrix between the insulator conditions and prediction conditions based on outcomes of the proposed indicators was performed in order to compare the performance of these indicators.

## Method and materials

### Test sample

**Figure 1:**
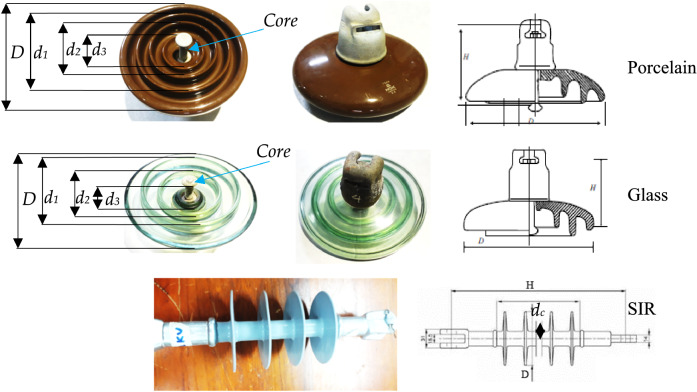
Insulators samples.

Three types of insulators specimens (porcelain, glass, and SIR) were collected from the transmission lines in Malaysia. The insulators were all naturally field-aged samples with similar aging and service histories. Figure 1 depicts the primary form of the chosen insulators. Table 1 lists the specifications of the insulators.

**Table 1:** Insulators parameters.

Parameter	Symbol	Porcelain	Glass	SIR
Leakage distance	Insulator length	32	34	40
High	H (cm)	14.6	14.6	33
Diameter	D(cm)	25.5	28	10
Rib diameters	d_1_(cm)	19.5	22.5	–
	d_2_(cm)	14.5	16	–
	d_3_(cm)	10.5	8	–
Core diameter	d_c_(cm)	5	5	8

### Experimental setup

**Figure 2:**
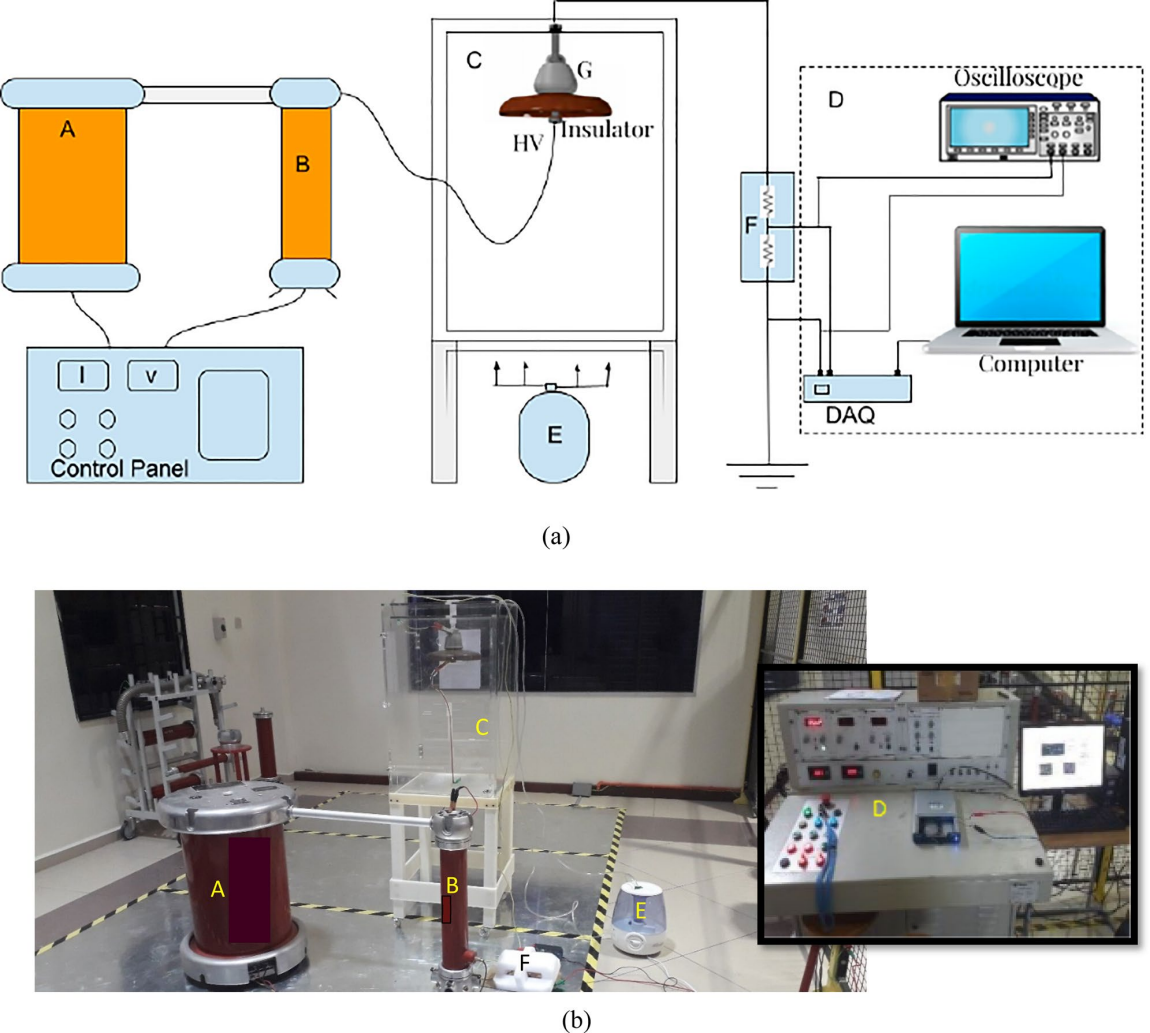
Insulator test: (**a**) Schematic diagram for experimental setup; (**b**) Test pictorial view.

The experimental setup was carried out according to the IEC 60,507 standard^35^ "Artificial pollution tests on high-voltage ceramic and glass insulators to be used in AC systems". All of the trials were conducted in a 50 × 50 × 75 cm test room with polycarbonate sheet walls. Four inlet valves were put in the chamber that was used to moisten the test insulators. Figure 2a shows the schematic diagram for the high voltage insulator experimental setup. Figure 2b depicts a visual representation of the high voltage laboratory's test setup and equipment. The following components made up the experimental circuit setup: A is an energized transformer (230 V/100 kV, 5 kVA, 50 Hz), B is a capacitive voltage divider, C is a test sample, D is a LC monitoring system, E is a steam fog generator and controller, and F is a resistive voltage divider (10,000:1) that is employed to capture the LC data. During the test, the energizing voltage was steadily increased by 2 kV step voltage until it reached 11 kV, and LC was measured at 11 kV. With 11 kV, LC was monitored without interruption under clean, mild, and medium pollution conditions. Under heavy pollution and wet condition, the step voltage was steadily increased by 1 kV to avoid unintended flashover; under this condition, the discharge activities and the flashover could happen before 11 kV. When a flashover occurred, LC rose sharply to about 10 times the critical current, thus interrupting the voltage supply system.

### Pollution and wetting process

Liquid alcohol was used to thoroughly remove traces of oil and grime from all insulators ahead of the experiment. The insulators were then let to dry naturally for one day under the sun's rays. After that, using the solid layer approach^36–38^, the contamination was placed on the insulator surface. The SDD was determined using sodium chloride (NaCl) salt while the NSDD was determined using kaolin. 1000 mg of water was used to evenly mix NaCl salt and kaolin uniformly. A conductivity meter was utilized to measure the pollutant solution conductivity at room temperature in order to determine SDD. The SDD was then estimated using Eq. (1) based on the IEC 60507 standard^35^:

1 $$SDD = (5.7 \times \sigma_{20} )^{1.03} \times V/A$$

where σ_20_ denotes the pollution solution conductivity at 20 °C, V denotes the pollution solution volume, and A is the insulator surface area. Meanwhile, Eq. (2) was used to determine the NSDD.

2 $$NSDD = ((w_{s} - w_{i} ) \times 10^{3} )/A$$

where *w*_*s*_ is the mass of the filter paper under pollution and *w*_*i*_ is the mass of the filter paper in dry conditions. As shown in Table 2, three degrees of SDD and NSDD were assessed in this study, corresponding to light, medium, and high pollution based on the IEC 60,507 standard^35^. The sample was artificially polluted and hung vertically in the test room for around 24 h to allow it to dry naturally. The test room pressure remained constant throughout the experiment, matching the laboratory's ambient pressure of about 99.5 kPa. The temperature in the test room was roughly 28 °C, which was about the same as the indoor temperature in Johor, Malaysia. The wetting procedure was carried out using the spray technique. To moisten the tested insulators, eight nozzles were placed around the chamber wall in a regular pattern. The control panel outside the high voltage test cage was in charge of controlling the fog flow rate. The controller was used to manage the wetting rate of the contaminated insulators by adjusting the water flow rate and air pressure. Three levels of wetting rates were employed to mimic the wetting of insulators in different climates: 3 l/h, 6 l/h, and 9 l/h corresponding to low, moderate, and high wetting, respectively^39^. An issue taken into consideration in choosing the wetting rate between 3 and 9 l/h was the minimum time to wet the sample with no hazard in the pollution layer on the insulator surface.

**Table 2:** Pollution severity readings.

Parameters	Values			
*σ*_*20*_ (S/m)	0.00	0.39	0.79	1.38
SDD (mg/cm^2^)	0.00	0.05	0.12	0.20
NSDD (mg/cm^2^)	0.00	0.15	0.25	0.35
Wt (l/h)	0	3	6	9
Contamination level	Clean	Light	Medium	Heavy

The insulators were tested under non-uniform and uniform pollutant scenarios. In the case of non-uniform contamination, three distinct contamination ratios of the top to bottom side SDD (P_u_/P_L_) were chosen: 1/3, 1/5, and 1/8. The insulator top and bottom surfaces were polluted separately during the non-uniform application of the contamination layer to generate SDD_1_ and SDD_2_, whereas the overall SDD may be met by Eq. (3)^5,40^:

3 $$SDD = \frac{{SDD_{1} \times S_{1} + SDD_{2} \times S_{2} }}{{S_{1} + S_{2} }}$$

where S1 and S2 are the insulator's upper and lower surface areas, respectively. Based on these specified pollution ratios, the pollution of the upper side SDD1 and lower side SDD2 can be satisfied by Eq. (4):

4 $$SDD_{1} = \frac{2 \times SDD}{{1 + (P_{u} /P_{L} )}},\quad SDD_{2} = \frac{2 \times SDD}{{1 + (P_{u} /P_{L} )}}$$

### Flashover voltage test

The flashover voltage testing was carried in the test chamber. The flashover voltage was measured using the up-and-down technique. For each contaminated sample, at least 11 tests were done. After passing the first acceptable experiment, the flashover voltage was changed by 5% lower or higher than the prior value in the next ten tests. As a result, the voltage was raised (or decreased) by 2 kV/s increments up (or down) to flashover. In the event of a flashover, the following test was conducted with a 5% lower voltage value; otherwise, the voltage was increased by 5% (after at least 20 s). The average flashover voltage (U_FO_) and standard deviation (σ) were determined using Eqs. (5 and 6), respectively:

5 $$U_{FO} = \sum\limits_{i = 1}^{N} {U_{i} } /N,\,\,\,\,\,\,\,\,\,N = 11$$

6 $$\sigma = \frac{{\sqrt {\sum\limits_{i = 1}^{N} {\left( {U_{i} - U_{FO} } \right)} } }}{N - 1} \times \frac{100}{{U_{FO} }}$$

where *U*_*i*_ represents the applied voltage in the *i*th test and *N* is the total number of tests.

### Monitoring of data

**Figure 3:**
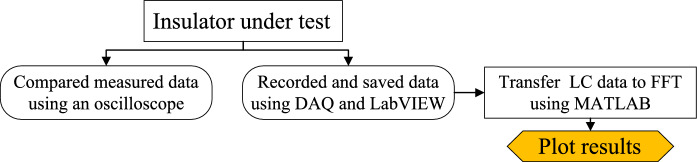
Leakage current monitoring process.

The applied voltage was measured using a capacitive voltage divider via the control panel in the experimental setup as shown in Fig. 2. Meanwhile, the LC monitoring system for LC includes a data acquisition (DAQ) card NI6024E, a computer, and an oscilloscope. A voltage downscaling divider (10,000:1) was employed since the DAQ's input voltage range was just 10 V. The data was sent from the DAQ to the computer, where it was stored as a CSV file and shown in Laboratory Virtual Instrument Engineering Workbench (LabVIEW). The oscilloscope was used to calibrate the DAQ data reading for testing the accuracy of the data reading. Matrix Laboratory (MATLAB) software was used to convert the LC signal in the time domain into the frequency domain. Figure 3 shows the technique used for monitoring and assessing LC.

### Characteristic parameter of leakage current

**Figure 4:**
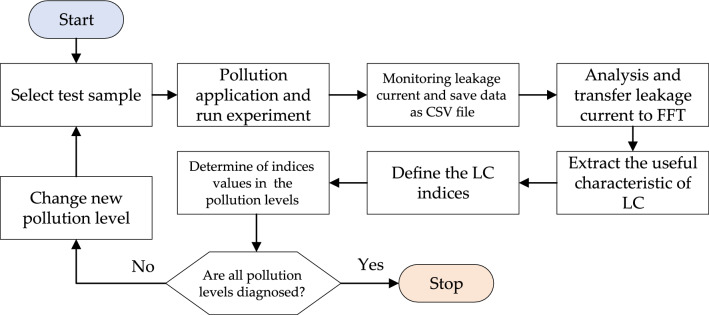
Flowchart process of insulator condition diagnosis.

One of the effective ways for developing new techniques for detecting insulators’ health is to extract features from LC waveform. These properties may be derived from the frequency and time domains of the LC signal. Six LC characteristics/indicators were extracted in both the time and frequency domains in this work. Figure 4 shows the insulator condition diagnostic diagram utilizing LC characteristics.

### Leakage current characteristic in time domain

Four characteristics were chosen in the temporal domain of LC. The peak of LC (*I*_*m*_) and the phase shift between applied voltage and LC (*ϕ*) were derived from the general formula of the AC shown in Eq. (7) ^41^:

7 $$I = I_{m} \sin (\omega t + \phi )$$

where ω is angular frequency calculated by, ω = 2πf, with the value of frequency f in this study being 50 Hz. So, the first two characteristics can be defined in Eqs. (8) and (9):

8 $$C_{1} = I_{m} \,$$

9 $$C_{2} = \phi = \frac{\Delta t}{T}360^\circ$$

**Figure 5:**
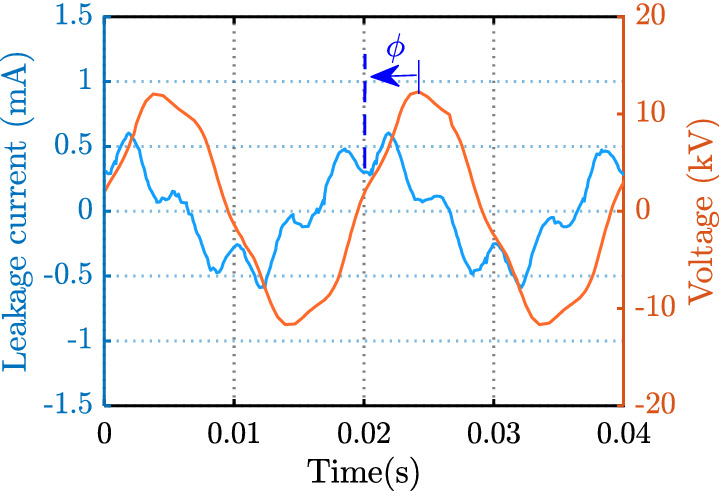
The phase difference between leakage current and applied voltage.

The phase difference between the applied voltage and LC (ϕ), as shown in Fig. 5, was determined using MATLAB software.

**Figure 6:**
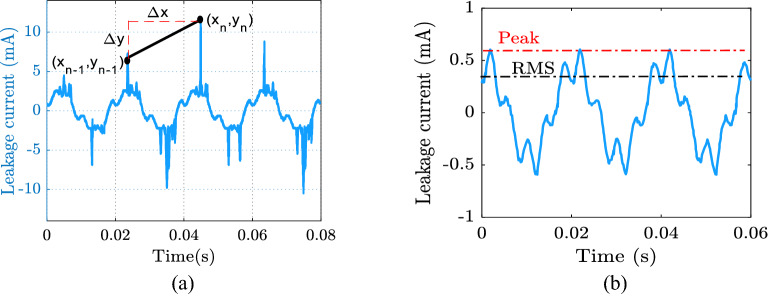
Extracting leakage current characteristics: (**a**) slope curve, (**b**) crest factor.

Calculating the slope of the line between two successive leakage current signal peaks yielded the third indication C_3_. As a result, Eq. (10) is used to represent C_3_:

10 $$C_{3} = \frac{{\sum\limits_{n = 1}^{m} {\left| {y_{n} - y_{n - 1} } \right|} }}{{x_{n} - x_{n - 1} }} = \frac{{\sum\limits_{0}^{m} {\left| {\Delta y_{n} } \right|} }}{{\Delta x_{n} }}$$

where *∆y*_*n*_ is the difference in leakage current between neighboring peaks at n, and *∆x*_*n*_ is the time period between these peaks. Figure 6a shows how the LC signal slope was determined. The fourth index was the crest factor (C_4_), which was calculated by dividing the peak value by the RMS value of the leakage current (as illustrated in Fig. 6b). As a result, Eq. (11) was used to represent C_4_:

11 $$C_{4} = \frac{{I_{peak} }}{{I_{RMS} }}$$

### Characteristic of the leakage current in frequency domain

The LC frequency domain at a frequency below 500 Hz for insulators under contamination has characteristic features. Therefore, in this work, the odd harmonics and total harmonic distortion (THD) of LC under 500 Hz were employed to propose indicators for insulators' condition assessment. The LC frequency features were defined by the C_5_ (THD) and C_6_ (harmonics ratio) indices in Eqs. (12 and 13), respectively:

12 $$C_{5} = THD\, = \frac{{\sqrt {\sum\limits_{n = 2}^{\infty } {I_{n} } } }}{{I_{1} }}\,\,\,\,\,\,\,$$

13 $$C_{6} = \frac{{\sum\limits_{n} {I_{n} } }}{{I_{3} }}\quad \quad n = 5,7,9$$

where *I*_*n*_ is the nth order harmonic and n represents the order odd harmonics number.

### Flashover voltage indicator

The flashover voltage indicator (C_7_) was defined as in Eq. (14):

14 $$C_{7} = \frac{{U_{FO} - U_{o} }}{{U_{o} }}\,\,\,\,\,\,\,\,\,\,\,$$

where U_o_ is the flashover voltage under clean conditions, which was measured to be around 53.4 kV for the porcelain insulator, 61.7 kV for the glass insulator and 66 kV for the SIR insulator.

## Results and discussion

### Leakage current results

**Figure 7:**
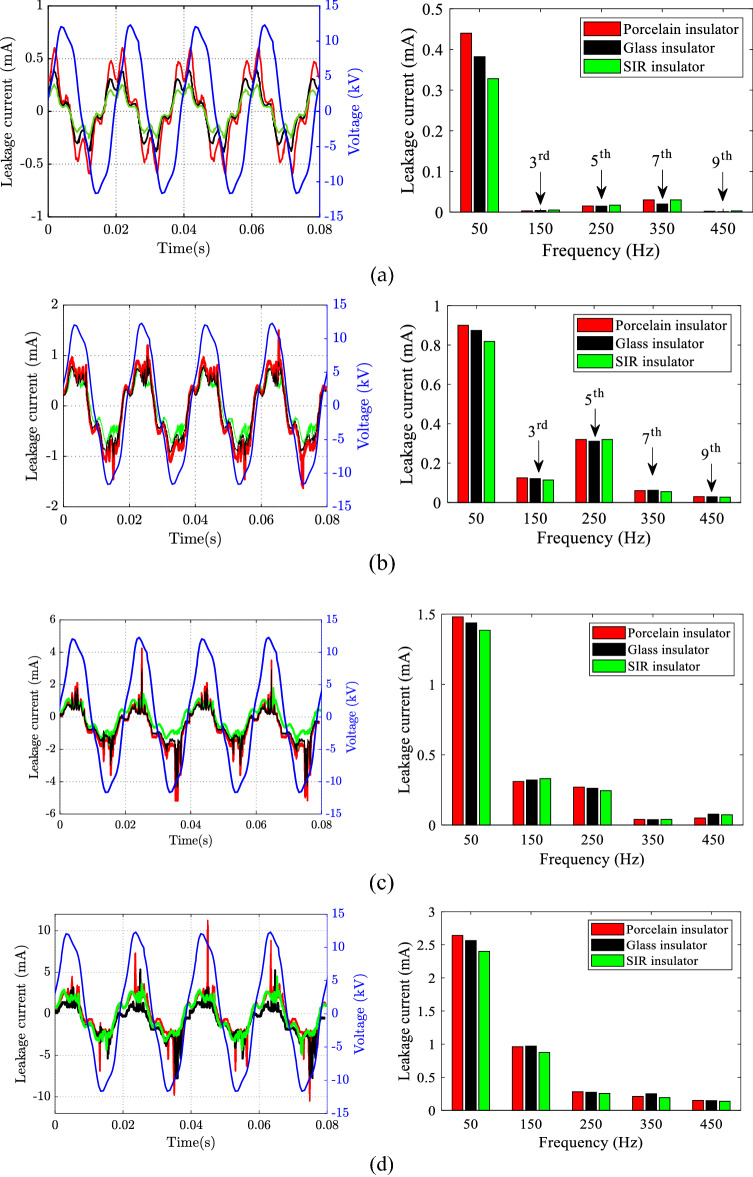
LC waveform and its FFT under different SDD pollution degree, 0.15 of NSDD, and 3 l/h of wetting rate: (**a**) SDD = 0.00 mg/cm^2^; (**b**) SDD = 0.05 mg/cm^2^, (**c**) SDD = 0.12 mg/cm^2^, (**d**) SDD = 0.2 mg/cm^2^.

**Figure 8:**
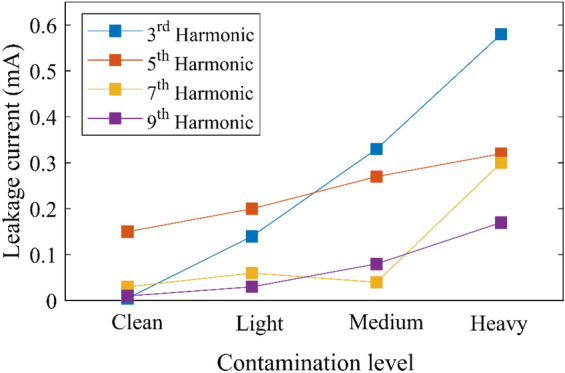
Odd harmonics of leakage current under pollution grading.

Figure 7 depicts the sample of LC results in the time and frequency domains for porcelain, glass, and SIR insulators under different SDD pollution levels, 0.15 mg/cm^2^ of NSDD and 3 l/h of Wt and uniform pollution distribution. Figure 7 illustrates that increasing pollution severity SDD under specific NSDD, Wt, and P_u_/P_L_ causes a large rise in LC. The current spike could be explained by the creation of a layer because of pollution and moisture, which enhanced conductivity along the insulator's surface. Consequently, a path was created to flow LC in the form of ions between insulator poles. Spot-arcing was found on occasion under high pollution conditions, particularly in the presence of moisture. The signal of LC looked to be substantially warped during the arcing activities, as illustrated in Fig. 7d. When LC increased, THD and harmonics levels also increased, but the phase shift angle between LC and voltage decreased. Because the resistive current increased with the constant capacitive current, the phase angle between LC and voltage decreased. Furthermore, a great variance in the odd harmonics 3rd to 9th could be observed when the contamination severity on the surfaces of the insulator was increased gradually, where the third harmonic would increase to overtake other odd harmonics (5th, 7th, and 9th), with a considerable rise in both of the 7th and 9th harmonics, as shown in Fig. 8. It can be noted that when the insulator had an arcing activity on its surface, the 3rd harmonic increased sharply.

Table 3 represents the characteristics of measured LC (*I*_*m*_, harmonics, THD, and *ϕ*) of tested insulators under various levels of uniform pollution for all the circumstances in Table 2. The LC characteristics under non-uniform pollution distribution are listed in Tables A1–A3 in Appendix A. The 5th and 7th harmonics components were bigger than the 3rd harmonic component in the clean state. Furthermore, this state showed no evidence of flashover. It could be observed that LC rose marginally as the wetting rate increased when the clean insulators were tested under various wetting conditions. This indicates that wetting the insulator surface causes increased charges to flow from the high voltage electrode to the ground electrode. Table 3 shows that LC on a clean insulator surface is very low, approximately 0.183 mA, and that it is primarily capacitive, with a phase change angle of around 90°. Tables A1–A3 in Appendix A demonstrate the effects of P_u_/P_L_, Wt, and NSDD. Generally, the test findings in Table 3 and Tables A1–A3 indicate that:

1. Surface conductivity was lowest in dry circumstances. As a result, raising SDD and NSDD had only a minor influence on LC and its components.
2. As the pollution levels SDD and NSDD, as well as the wetting rate, rose, LC increased significantly.
3. As SDD, NSDD, and Wt increased and P_u_/P_L_ dropped, LC harmonic amplitudes and THD increased. Meanwhile, *ϕ* decreased when SDD, NSDD, and Wt rose and P_u_/P_L_ dropped.

**Table 3:** Leakage current components for different pollution levels under uniform pollution distribution.

*SDD*	*NSDD*	Wt	Porcelain insulator							Glass insulator							Polymer insulator						
			*I*_*m*_	3rd	5th	7th	9th	THD	*ϕ*	*I*_*m*_	3rd	5th	7th	9th	THD	*ϕ*	*I*_*m*_	3rd	5th	7th	9th	THD	*ϕ*
0.00	0	0	0.183	0.0005	0.002	0	0.002	6.76	90	**0.162**	**0.0006**	**0.001**	**0.001**	**0.002**	**6.44**	**89.6**	0.158	0.0004	0.003	0	0.001	6.53	90
	0.15	0	0.20	0.0006	0.003	0.001	0.0005	6.82	90	**0.193**	**0.0005**	**0.002**	**0.001**	**0.0006**	**6.77**	**89.3**	0.187	0.0005	0.004	0.0007	0.0006	6.41	90
		3	0.44	0.003	0.0151	0.03	0.0008	7.43	87.2	**0.322**	**0.004**	**0.0147**	**0.02**	**0.0007**	**7.23**	**87.6**	0.268	0.005	0.017	0.03	0.0005	7.17	87.4
		6	0.72	0.004	0.0127	0.008	0.0007	7.52	87.13	**0.96**	**0.005**	**0.0125**	**0.006**	**0.001**	**7.48**	**87.17**	0.70	0.004	0.012	0.009	0.0004	7.46	87.11
		9	0.90	0.007	0.017	0.01	0.006	8.04	86.01	**0.84**	**0.007**	**0.022**	**0.007**	**0.004**	**7.85**	**85.73**	0.76	0.009	0.023	0.008	0.006	8.11	84.91
	0.25	0	0.22	0.003	0.014	0.002	0.002	6.95	88.98	**0.18**	**0.004**	**0.016**	**0.001**	**0.003**	**6.84**	**88.87**	0.16	0.002	0.011	0.003	0.001	6.78	88.91
		3	0.67	0.01	0.041	0.005	0.006	7.93	87.25	**0.60**	**0.008**	**0.038**	**0.004**	**0.004**	**7.87**	**87.11**	0.56	0.007	0.031	0.006	0.004	7.75	87.02
		6	0.87	0.0108	0.04	0.008	0.007	7.99	87.34	**0.83**	**0.012**	**0.03**	**0.01**	**0.005**	**7.72**	**87.03**	0.75	0.01	0.02	0.006	0.008	7.55	86.85
		9	0.97	0.017	0.06	0.01	0.008	8.38	86.45	**0.93**	**0.016**	**0.05**	**0.011**	**0.008**	**7.8**	**86.05**	0.88	0.015	0.05	0.008	0.009	8.12	86.14
	0.35	0	0.41	0.0058	0.012	0.009	0.009	7.31	88.26	**0.398**	**0.006**	**0.012**	**0.009**	**0.009**	**7.09**	**85.689**	0.373	0.005	0.011	0.008	0.008	6.645	80.236
		3	0.74	0.018	0.06	0.005	0.003	8.4	86.06	**0.718**	**0.017**	**0.058**	**0.005**	**0.003**	**8.15**	**83.553**	0.673	0.016	0.055	0.005	0.003	7.636	78.236
		6	0.91	0.02	0.0635	0.006	0.005	8.62	85.44	**0.883**	**0.019**	**0.062**	**0.006**	**0.005**	**8.36**	**82.951**	0.827	0.018	0.058	0.005	0.005	7.836	77.673
		9	1.05	0.033	0.073	0.007	0.009	8.73	85.04	**1.019**	**0.032**	**0.071**	**0.007**	**0.009**	**8.47**	**82.563**	0.955	0.030	0.066	0.006	0.008	7.936	77.309
0.05	0.15	0	0.522	0.009	0.043	0.009	0.004	8.21	82.2	**0.507**	**0.009**	**0.042**	**0.009**	**0.004**	**7.97**	**79.806**	0.475	0.008	0.039	0.008	0.004	7.464	74.727
		3	0.9	0.125	0.32	0.06	0.03	10.23	79.31	**0.874**	**0.121**	**0.311**	**0.062**	**0.029**	**9.93**	**77.000**	0.818	0.114	0.32	0.055	0.027	9.300	72.100
		6	1.19	0.136	0.3	0.083	0.04	11.29	67.5	**1.155**	**0.132**	**0.291**	**0.081**	**0.039**	**10.961**	**65.534**	1.082	0.124	0.273	0.075	0.036	10.264	61.364
		9	1.57	0.18	0.36	0.092	0.077	14.42	57.61	**1.524**	**0.175**	**0.350**	**0.089**	**0.075**	**14.000**	**55.932**	1.427	0.164	0.327	0.084	0.070	13.109	52.373
	0.25	0	0.73	0.014	0.056	0.008	0.007	8.901	80.75	**0.709**	**0.014**	**0.054**	**0.008**	**0.007**	**8.642**	**78.398**	0.664	0.013	0.051	0.007	0.006	8.092	73.409
		3	1.23	0.142	0.231	0.085	0.066	10.89	66.21	**1.194**	**0.138**	**0.224**	**0.083**	**0.064**	**10.573**	**64.282**	1.118	0.129	0.210	0.077	0.060	9.900	60.191
		6	1.49	0.167	0.23	0.14	0.07	12.85	54.25	**1.447**	**0.162**	**0.223**	**0.136**	**0.068**	**12.47**	**52.670**	1.355	0.152	0.209	0.127	0.064	11.682	49.318
		9	1.84	0.179	0.231	0.15	0.08	15.03	42.66	**1.786**	**0.174**	**0.224**	**0.146**	**0.078**	**14.59**	**41.417**	1.673	0.163	0.210	0.136	0.073	13.664	38.782
	0.35	0	0.91	0.018	0.065	0.013	0.01	9.631	78.32	**0.883**	**0.017**	**0.063**	**0.013**	**0.010**	**9.350**	**76.039**	0.827	0.016	0.059	0.012	0.009	8.755	71.200
		3	1.63	0.19	0.252	0.102	0.056	13.34	56.21	**1.583**	**0.184**	**0.245**	**0.099**	**0.054**	**12.95**	**54.573**	1.482	0.173	0.229	0.093	0.051	12.127	51.100
		6	2.04	0.22	0.24	0.14	0.09	15.77	40.13	**1.981**	**0.214**	**0.233**	**0.136**	**0.087**	**15.31**	**38.961**	1.855	0.200	0.218	0.127	0.082	14.336	36.482
		9	2.61	0.261	0.32	0.12	0.11	18.84	33.22	**2.534**	**0.253**	**0.311**	**0.117**	**0.107**	**18.29**	**32.252**	2.373	0.237	0.291	0.109	0.100	17.127	30.200
0.12	0.15	0	0.636	0.022	0.072	0.011	0.008	8.21	71.2	**0.617**	**0.021**	**0.070**	**0.011**	**0.008**	**7.971**	**69.126**	0.578	0.020	0.065	0.010	0.007	7.464	64.727
		3	1.48	0.31	0.27	0.04	0.05	15.33	55.21	**1.437**	**0.320**	**0.262**	**0.039**	**0.078**	**14.883**	**53.602**	1.385	0.33	0.245	0.04	0.073	13.936	50.191
		6	1.63	0.41	0.32	0.062	0.12	18.88	38.53	**1.583**	**0.398**	**0.311**	**0.060**	**0.117**	**18.330**	**37.408**	1.482	0.373	0.291	0.056	0.109	17.164	35.027
		9	1.93	0.46	0.41	0.095	0.08	23.25	27.16	**1.874**	**0.447**	**0.398**	**0.092**	**0.078**	**22.573**	**26.369**	1.755	0.418	0.373	0.086	0.073	21.136	24.691
	0.25	0	0.75	0.032	0.084	0.013	0.021	9.88	75.89	**0.728**	**0.031**	**0.082**	**0.013**	**0.020**	**9.592**	**73.680**	0.682	0.029	0.076	0.012	0.019	8.982	68.991
		3	1.72	0.35	0.31	0.104	0.09	21.74	52.52	**1.670**	**0.340**	**0.301**	**0.101**	**0.087**	**21.107**	**50.990**	1.564	0.318	0.282	0.095	0.082	19.764	47.745
		6	1.96	0.53	0.37	0.11	0.098	25.25	30.22	**1.903**	**0.515**	**0.359**	**0.107**	**0.095**	**24.515**	**29.340**	1.782	0.482	0.336	0.100	0.089	22.955	27.473
		9	2.34	0.57	0.41	0.13	0.11	31.97	25.12	**2.272**	**0.553**	**0.398**	**0.126**	**0.107**	**31.039**	**24.388**	2.127	0.518	0.373	0.118	0.100	29.064	22.836
	0.35	0	0.94	0.041	0.088	0.031	0.023	9.91	70.43	**0.913**	**0.040**	**0.085**	**0.030**	**0.022**	**9.621**	**68.379**	0.855	0.037	0.080	0.028	0.021	9.009	64.027
		3	1.98	0.56	0.36	0.13	0.16	30.51	39.66	**1.922**	**0.544**	**0.350**	**0.126**	**0.155**	**29.621**	**38.505**	1.800	0.509	0.327	0.118	0.145	27.736	36.055
		6	2.04	0.72	0.41	0.15	0.13	34.63	20.63	**1.981**	**0.699**	**0.398**	**0.146**	**0.126**	**33.621**	**20.029**	1.855	0.655	0.373	0.136	0.118	31.482	18.755
		9	2.61	0.62	0.42	0.17	0.18	42.04	14.95	**2.534**	**0.602**	**0.408**	**0.165**	**0.175**	**40.816**	**14.515**	2.373	0.564	0.382	0.155	0.164	38.218	13.591
0.2	0.15	0	0.842	0.053	0.096	0.045	0.032	9.92	56.84	**0.817**	**0.051**	**0.093**	**0.044**	**0.031**	**9.631**	**55.184**	0.765	0.048	0.087	0.041	0.029	9.018	51.673
		3	2.64	0.96	0.28	0.21	0.15	36.56	32.12	**2.563**	**0.97**	**0.272**	**0.25**	**0.146**	**35.495**	**31.184**	2.400	0.873	0.255	0.191	0.136	33.236	29.200
		6	2.94	0.99	0.23	0.26	0.11	41.32	17.58	**2.854**	**0.961**	**0.223**	**0.252**	**0.107**	**40.117**	**17.068**	2.673	0.900	0.209	0.236	0.100	37.564	15.982
		9	3.65	1.087	0.23	0.27	0.13	43.77	11.56	**3.544**	**1.055**	**0.223**	**0.262**	**0.126**	**42.495**	**11.223**	3.318	0.988	0.209	0.245	0.118	39.791	10.509
	0.25	0	1.03	0.062	0.1	0.063	0.032	10.78	42.54	**1.000**	**0.060**	**0.097**	**0.061**	**0.031**	**10.466**	**41.301**	0.936	0.056	0.091	0.057	0.029	9.800	38.673
		3	2.93	0.95	0.28	0.21	0.11	45.63	26.45	**2.845**	**0.922**	**0.272**	**0.204**	**0.107**	**44.301**	**25.680**	2.664	0.864	0.255	0.191	0.100	41.482	24.045
		6	3.29	1.09	0.27	0.23	0.12	47.84	10.73	**3.194**	**1.058**	**0.262**	**0.223**	**0.117**	**46.447**	**10.417**	2.991	0.991	0.245	0.209	0.109	43.491	9.755
		9	3.99	1.3	0.32	0.24	0.12	53.79	1.04	**3.874**	**1.262**	**0.311**	**0.233**	**0.117**	**52.223**	**1.010**	3.627	1.182	0.291	0.218	0.109	48.900	0.945
	0.35	0	1.07	0.065	0.102	0.048	0.05	10.63	32.84	**1.039**	**0.063**	**0.099**	**0.047**	**0.049**	**10.320**	**31.883**	0.973	0.059	0.093	0.044	0.045	9.664	29.855
		3	3.54	1.392	0.3	0.12	0.15	51.67	3.92	**3.437**	**1.351**	**0.291**	**0.117**	**0.146**	**50.165**	**3.806**	3.218	1.265	0.273	0.109	0.136	46.973	3.564
		6	5.24	1.62	0.35	0.21	0.12	59.29	2.07	**5.087**	**1.573**	**0.340**	**0.204**	**0.117**	**57.563**	**2.010**	4.764	1.473	0.318	0.191	0.109	53.900	1.882
		9	6.51	1.79	0.27	0.13	0.17	60.86	0.83	**6.320**	**1.738**	**0.262**	**0.126**	**0.165**	**59.087**	**0.806**	5.918	1.627	0.245	0.118	0.155	55.327	0.755

Significant values are in [bold].

### Flashover voltage results

**Figure 9:**
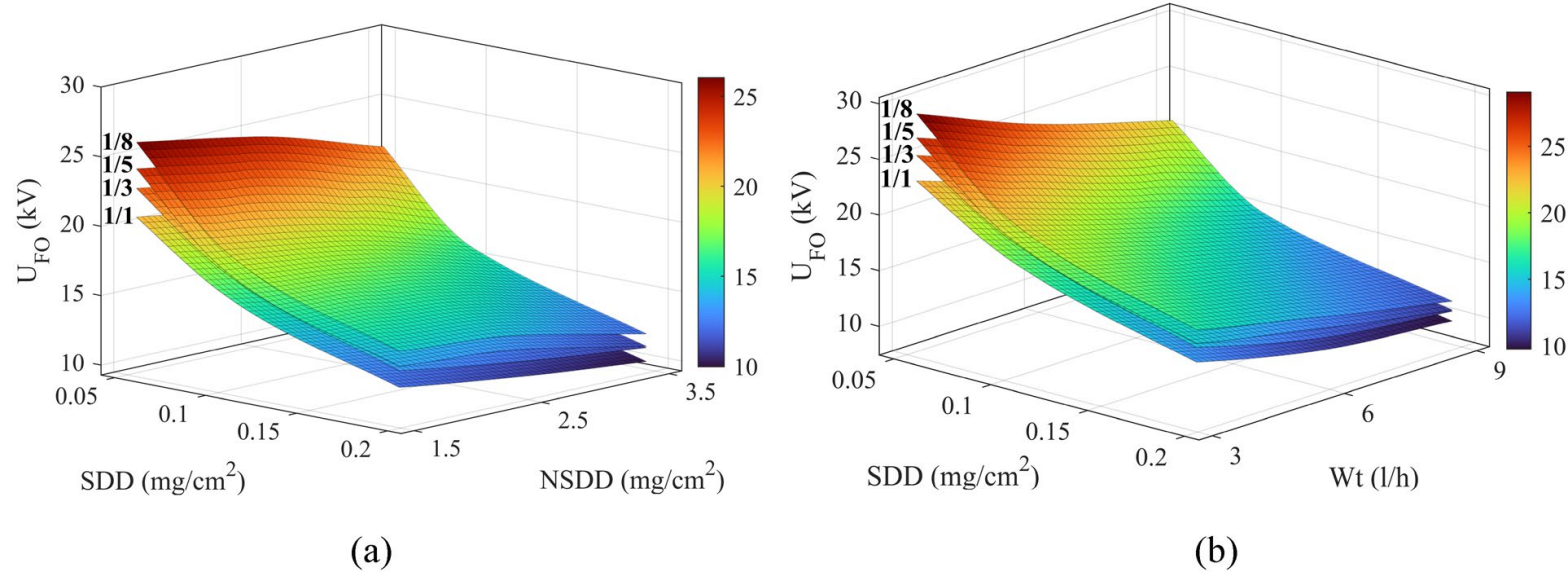
Flashover voltage of glass insulator vs. (**a**) SDD and NSDD (**b**) SDD and Wt under different non-uniformity degrees P_u_/P_L_.

The flashover voltage experimental result under different conditions for the tested insulators are listed in Table 4. The flashover voltage as a function of SDD and NSDD under different P_u_/P_L_ for the glass specimen as an example is displayed in Fig. 9a. Meanwhile, Fig. 9b depicts the influence of SDD and wetting rate on the flashover voltage. The findings indicate that the flashover voltage drops significantly with the rising value of the pollution severity/SDD. As the contamination severity increases, the contamination layer's electric conductivity improves. Therefore, the flashover takes place at a lower voltage level. According to the results in Table 4 and Fig. 9, it can be noted that the effect of wetting rate on the flashover voltage is greater than NSDD for the same values of SDD and Pu/PL.

**Table 4:** Flashover experimental results under different conditions for porcelain, glass, and SIR insulators.

Insulator type			Porcelain			Glass			SIR		
P_u_/P_L_	NSDD	SDD	Wt								
			3.00	6.00	9.00	3.00	6.00	9.00	3.00	6.00	9.00
1/1	0.15	0.05	24.12	19.61	18.33	25.76	20.95	19.58	28.65	23.29	21.77
		0.12	18.89	15.36	14.35	20.18	16.40	15.33	22.43	18.24	17.05
		0.2	13.81	11.23	10.49	14.75	11.99	11.21	16.40	13.33	12.46
	0.25	0.05	21.97	18.31	16.55	23.46	19.55	17.67	26.09	21.74	19.65
		0.12	17.20	13.80	12.00	18.37	14.74	12.82	20.43	16.39	14.25
		0.2	12.57	10.08	9.02	13.43	10.77	9.63	14.93	11.97	10.71
	0.35	0.05	20.58	16.73	15.64	21.98	17.87	16.70	24.44	19.87	18.57
		0.12	15.52	12.62	11.79	16.57	13.47	12.59	18.43	14.98	14.00
		0.2	11.34	9.22	8.61	12.11	9.84	9.20	13.46	10.94	10.23
1/3	0.15	0.05	26.53	21.57	20.16	28.34	23.04	21.53	31.51	25.62	23.94
		0.12	20.78	16.89	15.79	22.19	18.04	16.86	24.68	20.06	18.75
		0.2	15.19	12.35	11.54	16.22	13.19	12.33	18.04	14.67	13.71
	0.25	0.05	24.16	20.14	17.08	25.80	21.51	18.24	28.69	23.91	20.28
		0.12	18.92	15.18	13.11	20.21	16.22	14.00	22.47	18.03	15.57
		0.2	13.83	11.09	9.83	14.77	11.85	10.50	16.43	13.17	11.67
	0.35	0.05	22.64	18.40	17.20	24.18	19.66	18.37	26.88	21.86	20.43
		0.12	17.07	13.88	12.97	18.23	14.82	13.85	20.27	16.48	15.40
		0.2	12.47	10.14	9.47	13.32	10.83	10.12	14.81	12.04	11.25
1/5	0.15	0.05	28.24	22.96	21.46	30.16	24.52	22.92	33.54	27.27	25.48
		0.12	20.94	17.03	15.91	22.37	18.18	16.99	24.87	20.22	18.90
		0.2	15.55	12.64	11.81	16.61	13.50	12.62	18.47	15.01	14.03
	0.25	0.05	25.72	21.53	18.64	27.46	23.00	19.91	30.54	25.57	22.14
		0.12	19.07	15.81	12.93	20.37	16.89	13.81	22.65	18.78	15.36
		0.2	14.16	11.92	9.91	15.12	12.73	10.58	16.81	14.16	11.77
	0.35	0.05	23.52	19.12	17.87	25.12	20.42	19.08	27.93	22.71	21.22
		0.12	16.82	13.67	12.78	17.96	14.60	13.65	19.97	16.24	15.18
		0.2	12.58	10.23	9.56	13.43	10.92	10.21	14.94	12.15	11.35
1/8	0.15	0.05	30.50	24.80	23.17	32.57	26.48	24.75	36.22	29.45	27.52
		0.12	22.62	18.39	17.19	24.16	19.64	18.35	26.86	21.84	20.41
		0.2	16.79	13.65	12.76	17.93	14.58	13.63	19.94	16.21	15.15
	0.25	0.05	27.77	23.25	19.99	29.66	24.84	21.35	32.98	27.62	23.74
		0.12	20.60	17.08	13.89	22.00	18.24	14.83	24.46	20.28	16.50
		0.2	15.29	12.88	10.62	16.33	13.75	11.35	18.16	15.29	12.62
	0.35	0.05	25.40	20.65	19.30	27.13	22.05	20.61	30.16	24.52	22.92
		0.12	18.16	14.77	13.80	19.40	15.77	14.74	21.57	17.54	16.39
		0.2	13.43	10.06	8.963	15.12	12.31	9.62	16.83	14.28	10.36

### Leakage current indicators trend under clean condition

As mentioned before, the value of LC changed minorly when the rate of wetting increased on a clean insulator; correspondingly, the LC characteristics would change. Accordingly, the LC indicators of clean insulators under different wetting rates have a slight change as shown in Fig. 9. It can be noted that there is no significant difference between different NSDD in the same wetting rate Wt in the clean condition. Generally, the indicators C_1_, C_3_, C_4_, and C_5_ increased with an increase in Wt and NSDD. In a clean state, when Wt increased from 3 to 9 l/h under NSDD of 0.15 mg/cm^2^, C_1_ increased from 0.42 to 0.83, C_3_ increased from 0.065 to 0.11, C_4_ increased from 1.56 to 1.585, and C_5_ increased from 0.0028 to 0.0049. On the contrary, the C_2_ and C_6_ decreased with the rise in NSDD and Wt. When the Wt increased from 3 to 9 l/h under a 0.15 mg/cm^2^ of NSDD, the C_2_ decreased from 87.2 to 86.01% and C_6_ decreased from 5.9 to 4.7. The slope of increment and decrement under NSDD 0.25 mg/cm^2^ and 0.35 mg/cm^2^ is approximately the same as in the 0.15 mg /cm^2^ with a slight change in the amplitude of indices.

### Leakage current indicators trend under various SDD

**Figure 10:**
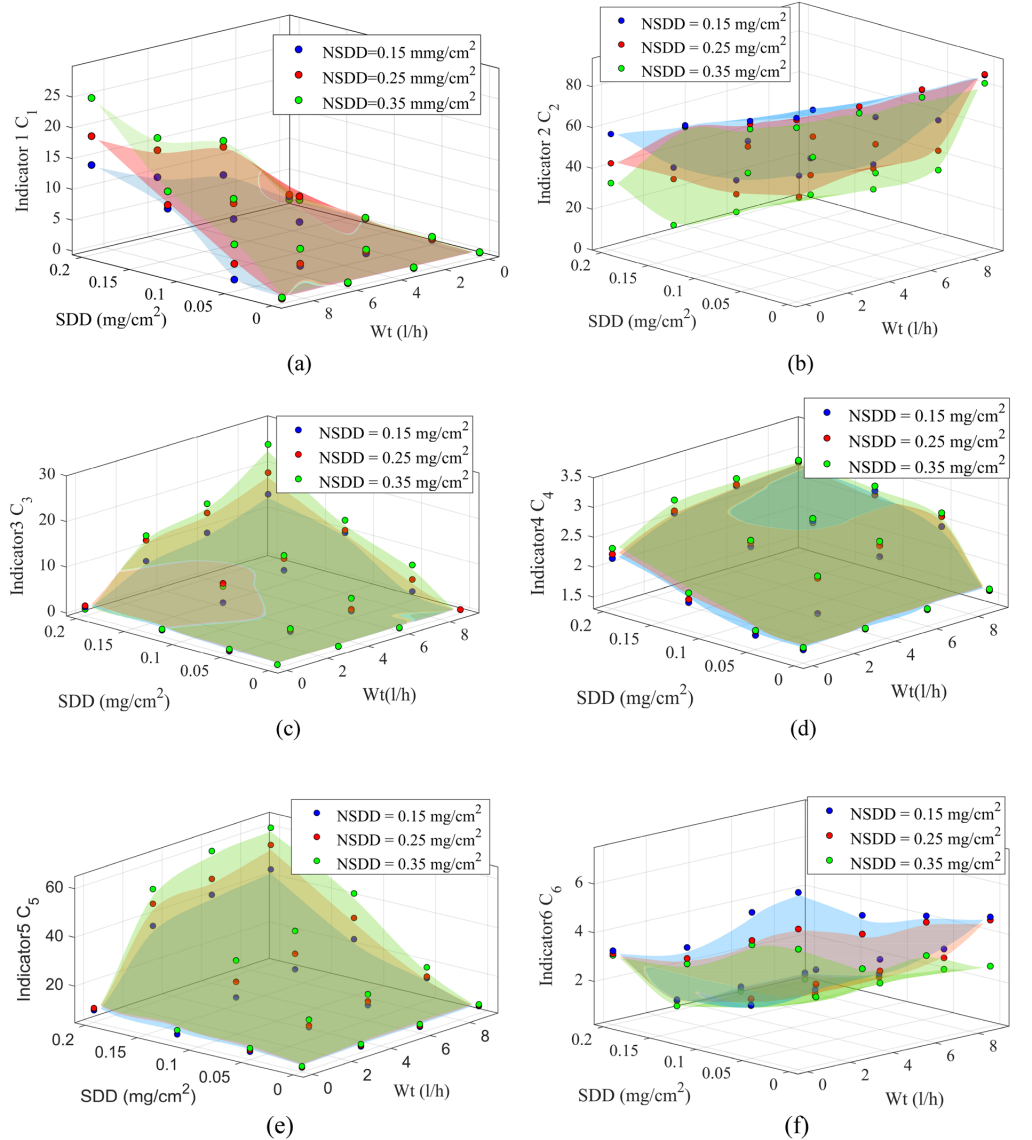
Leakage current indicators of uniform polluted insulators under various Wt and NSDD: (**a**) C_1_; (**b**) C_2_; (**c**) C_3_; (**d**) C_4_; (**e**) C_5_; (**f**) C_6_.

The SDD has a great effect on the LC indicators. The LC indicators C_1_, C_3_, C_4_, and C_5_ rose considerably when SDD increased under any NSDD, Wt, and P_u_/P_L_ while the C_2_ and C_6_ dropped dramatically as the SDD increased under the same conditions. To show the LC indicators trend under various SDD for porcelain insulator polluted uniformly, Fig. 10 was plotted. In addition, Table 5 illustrates the results of the LC indicators for the porcelain insulator as an example while the LC indicators result for both glass and SIR insulators are listed in Appendix B1 and B2. According to the results in Fig. 10 and Table 5, with C_1_ and C_6_ taken as an example, under NSDD of 0.25 mg/cm^2^, Wt of 6 l/h and P_u_/P_L_ of 1/1, C_1_ corresponded to 1.49, 1.96, and 3.29 mA when SDD was 0.05, 0.12, and 0.2 mg/cm^2^, respectively. It can be observed that the C_1_ increased by 10.4% and 15.95% when the SDD increased from 0.05 to 0.12 and 0.2 mg/cm^2^, respectively. Whereas for C_6_, when SDD was 0.05, 0.12 and 0.2 mg/cm^2^, C_6_ corresponded to 2.63, 1.09, and 0.56 mA, respectively. It can be said that the direct relationship of insulator surface conductivity with the SDD, which affects the LC signal, is the cause of the large influence on the LC indicators with changes in SDD.

**Table 5:** LC indicators under various SDD, wetting rate Wt and NSDD for non-uniform polluted porcelain insulator.

Pu/PL			*1/3*						*1/5*						*1/8*					
SDD mg/cm^2^	NSDD mg/cm^2^	W_t_ l/h	*C*_*1*_	*C*_*2*_	*C*_*3*_	*C*_*4*_	*C*_*5*_	*C*_*6*_	*C*_*1*_	*C*_*2*_	*C*_*3*_	*C*_*4*_	*C*_*5*_	*C*_*6*_	*C*_*1*_	*C*_*2*_	*C*_*3*_	*C*_*4*_	*C*_*5*_	*C*_*6*_
0.05	0.15	0	0.44	88.48	0.15	1.49	8.33	7.27	**0.39**	**90.25**	**0.14**	**1.49**	**8.29**	**8.80**	0.36	90.52	0.14	1.48	8.20	9.39
		3	0.76	85.37	0.39	1.59	10.38	3.57	**0.66**	**87.07**	**0.38**	**1.59**	**10.32**	**3.69**	0.62	87.33	0.38	1.58	10.22	3.89
		6	1.00	72.66	0.82	1.63	11.45	3.32	**0.88**	**74.11**	**0.80**	**1.62**	**11.40**	**3.50**	0.82	74.33	0.80	1.62	11.28	3.67
		9	1.33	62.01	1.08	1.64	14.63	3.18	**1.16**	**63.25**	**1.07**	**1.64**	**14.56**	**3.21**	1.08	63.44	1.06	1.63	14.40	3.61
	0.25	0	0.63	86.91	0.31	1.65	9.03	7.15	**0.54**	**88.66**	**0.31**	**1.65**	**8.99**	**8.23**	0.51	88.92	0.31	1.64	8.89	8.59
		3	1.06	71.27	0.72	1.62	11.05	2.95	**0.92**	**72.69**	**0.72**	**1.62**	**10.99**	**3.09**	0.86	72.91	0.71	1.61	10.88	3.22
		6	1.27	58.39	1.12	1.63	13.04	2.81	**1.12**	**59.57**	**1.11**	**1.63**	**12.97**	**2.93**	1.05	59.74	1.10	1.62	12.83	3.16
		9	1.58	45.92	3.23	1.65	15.25	2.79	**1.78**	**46.84**	**2.21**	**1.64**	**15.17**	**2.96**	1.29	46.97	3.18	1.64	15.02	2.96
	0.35	0	0.77	84.30	0.54	1.66	9.77	6.53	**0.68**	**85.98**	**0.54**	**1.66**	**9.72**	**7.75**	0.63	86.24	0.53	1.65	9.62	8.67
		3	1.39	60.51	0.91	1.63	13.53	2.28	**1.21**	**61.71**	**0.91**	**1.62**	**13.47**	**2.47**	1.13	61.90	0.90	1.62	13.32	2.59
		6	1.73	43.19	3.17	1.64	16.00	2.30	**1.52**	**44.06**	**3.16**	**1.64**	**15.92**	**2.52**	1.41	44.19	3.12	1.63	15.76	2.64
		9	2.23	35.76	5.88	1.65	19.11	2.22	**1.93**	**36.47**	**5.85**	**1.65**	**19.02**	**2.40**	1.81	36.59	5.80	1.65	18.82	2.51
0.12	0.15	0	0.54	76.64	0.66	1.67	8.33	5.58	**0.47**	**78.17**	**0.65**	**1.67**	**8.29**	**6.57**	0.44	78.41	0.65	1.66	8.20	8.23
		3	1.25	59.43	2.35	1.60	15.55	1.36	**1.10**	**60.61**	**2.34**	**1.60**	**15.48**	**1.69**	1.02	60.80	2.32	1.59	15.31	2.04
		6	1.39	41.48	4.90	1.66	19.15	1.39	**1.21**	**42.30**	**4.88**	**1.65**	**19.06**	**1.57**	1.13	42.43	4.83	1.65	18.86	1.76
		9	1.64	29.23	8.45	2.32	23.59	1.55	**1.43**	**29.81**	**8.40**	**2.32**	**23.47**	**1.60**	1.34	29.91	8.33	2.31	23.23	1.79
	0.25	0	0.62	81.69	0.82	2.53	10.02	5.29	**0.53**	**83.32**	**0.82**	**2.53**	**9.97**	**5.99**	0.50	83.57	0.80	2.52	9.86	7.68
		3	1.41	56.53	5.88	1.67	22.06	1.50	**1.23**	**57.66**	**5.85**	**1.67**	**21.95**	**1.63**	1.15	57.83	5.80	1.66	21.72	1.71
		6	1.61	32.53	7.05	2.27	25.61	1.37	**1.40**	**33.18**	**7.01**	**2.27**	**25.49**	**1.55**	1.31	33.27	6.95	2.26	25.23	1.62
		9	1.83	27.03	8.97	2.52	32.44	1.37	**1.60**	**27.58**	**8.91**	**2.52**	**32.28**	**1.63**	1.49	27.66	8.83	2.51	31.94	1.67
	0.35	0	0.79	75.80	0.83	2.70	10.05	4.35	**0.69**	**77.32**	**0.83**	**2.70**	**10.00**	**4.91**	0.65	77.56	0.82	2.69	9.90	6.17
		3	1.67	42.69	5.38	1.69	30.95	1.21	**1.46**	**43.55**	**5.36**	**1.68**	**30.80**	**1.43**	1.36	43.67	5.31	1.68	30.48	1.52
		6	1.72	22.21	7.68	2.31	35.13	1.21	**1.50**	**22.65**	**7.64**	**2.31**	**34.96**	**1.34**	1.40	22.72	7.57	2.30	34.59	1.59
		9	2.20	16.09	10.82	2.59	42.66	1.25	**1.92**	**16.42**	**10.75**	**2.59**	**42.44**	**1.29**	1.80	16.46	10.66	2.58	42.00	1.47
0.2	0.15	0	0.71	61.18	1.08	2.77	10.06	3.57	**0.60**	**62.41**	**1.07**	**2.76**	**10.01**	**3.95**	0.56	62.60	1.06	2.75	9.91	4.93
		3	2.24	34.57	6.14	1.84	37.10	0.70	**1.94**	**35.27**	**6.11**	**1.84**	**36.91**	**0.79**	1.82	35.37	6.05	1.84	36.52	0.95
		6	2.49	18.92	7.97	2.49	41.93	0.66	**2.16**	**19.30**	**7.92**	**2.49**	**41.72**	**0.69**	2.03	19.35	7.85	2.48	41.28	0.83
		9	3.08	12.45	11.78	2.62	44.41	0.63	**2.69**	**12.69**	**11.71**	**2.62**	**44.18**	**0.60**	2.52	12.73	11.60	2.61	43.72	0.75
	0.25	0	0.87	45.79	1.32	2.82	10.94	3.43	**0.76**	**46.70**	**1.32**	**2.81**	**10.88**	**3.74**	0.71	46.85	1.30	2.80	10.76	4.50
		3	2.48	28.47	9.99	1.90	46.29	0.74	**2.16**	**29.04**	**9.94**	**1.89**	**46.06**	**0.75**	2.02	29.12	9.84	1.89	45.58	0.79
		6	2.78	11.55	11.67	2.57	48.54	0.66	**2.42**	**11.78**	**11.60**	**2.56**	**48.30**	**0.74**	2.27	11.82	11.50	2.55	47.79	0.74
		9	3.38	1.12	15.73	2.58	54.57	0.56	**2.94**	**1.14**	**15.63**	**2.58**	**54.30**	**0.61**	2.75	1.15	15.49	2.57	53.73	0.68
	0.35	0	0.91	35.35	0.66	2.75	10.78	3.35	**0.78**	**36.05**	**0.66**	**2.75**	**10.73**	**3.64**	0.74	36.17	0.65	2.73	10.62	4.23
		3	3.19	4.22	10.83	2.02	52.43	0.59	**2.92**	**4.31**	**10.76**	**2.01**	**52.17**	**0.67**	1.75	4.32	10.66	2.01	51.61	0.82
		6	4.83	2.23	13.34	2.60	60.15	0.70	**3.44**	**2.27**	**13.27**	**2.60**	**59.86**	**0.60**	2.24	2.28	13.15	2.59	59.23	0.70
		9	5.51	0.00	20.96	2.66	61.75	0.48	**4.80**	**0.00**	**19.80**	**2.65**	**61.44**	**0.55**	4.48	0.00	17.51	2.64	60.80	0.63

Significant values are in [bold].

### Leakage current indicators trend under different NSDD

**Figure 11:**
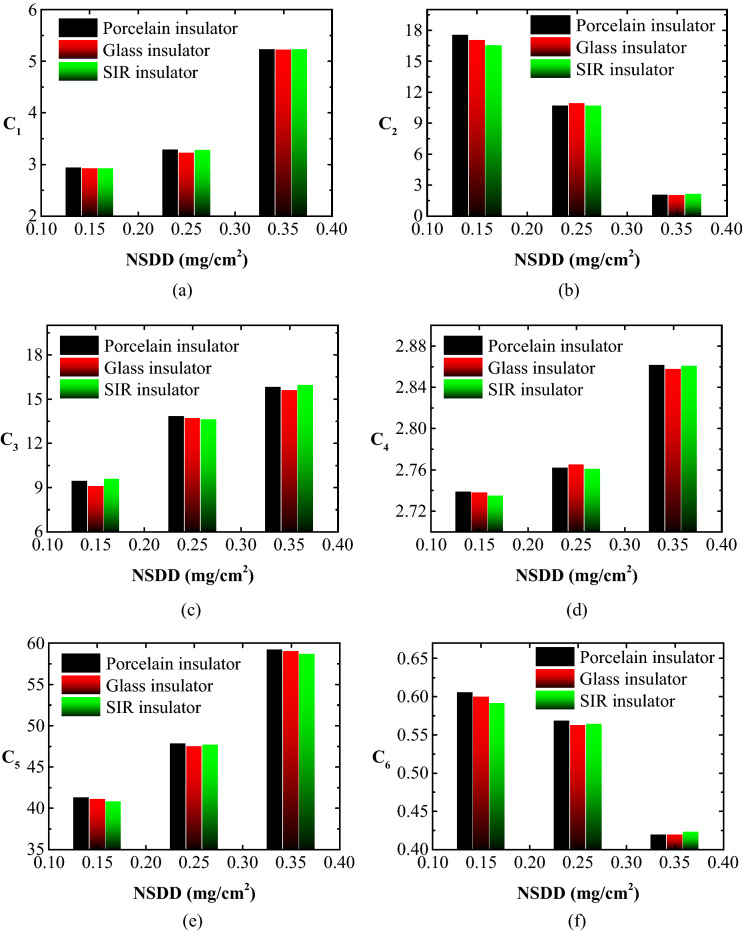
LC indicators versus NSDD for tested insulators under SDD = 0.2 mg/cm^2^, Wt = 9 l/h and P_u_/P_L_ = 1/1: (**a**) C_1_; (**b**) C_2_; (**c**) C_3_; (**d**) C_4_; (**e**) C_5_; (**f**) C_6_.

The performance of the indices is similar to the previous case with changing NSDD. However, there are discrepancies in the magnitude of increase or decrement. The test results indicated that for constant SDD, Wt, and P_u_/P_L_, if the NSDD increased, the C_1_, C_3_, C_4_, and C_5_ would increase while the C_2_ and C_6_ would decrease. Figure 11 depicts the C_1_, C_3_, C_4_, C_5_, and C_6_ versus NSDD curves for porcelain, glass, and SIR insulators with SDD = 0.2 mg/cm^2^, Wt = 6 l/h, and P_u_/P_L_ = 1/1 to help demonstrate the relationship between NSDD and the suggested indicators.

### Leakage current indicators trend under different Wt

**Figure 12:**
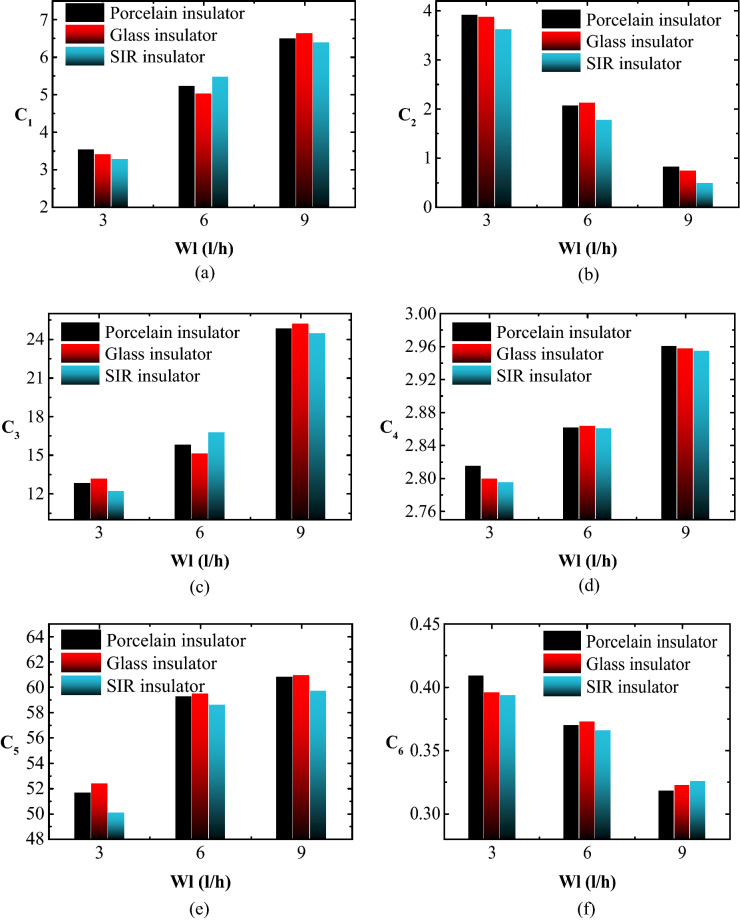
LC indicators versus Wt for tested insulators under SDD = 0.2 mg/cm^2^, NSDD = 0.35 mg/cm^2^ and P_u_/P_L_ = 1/1: (**a**) C_1_; (**b**) C_2_; (**c**) C_3_; (**d**) C_4_; (**e**) C_5_; (**f**) C_6_.

Figure 12 shows the connection between the suggested C_1_-C_6_ indices and Wt for porcelain, glass, and SIR insulators under SDD = 0.2 mg/cm^2^, NSDD = 0.35 mg/cm^2^, and P_u_/P_L_ = 1/1. It can be seen that when Wt increases, C_2_, and C_6_ decreases while the C_1_, C_3_, C_4_, and C_5_ increases. For example, when Wt increased from 3 to 6 and 9 l/h with SDD = 0.2 mg/cm^2^, NSDD = 0.35 mg/cm^2^, and P_u_/P_L_ = 1/1, C_1_ for porcelain insulator rose by 13.4% and 15.4%, respectively. Meanwhile, C_2_ fell by 72.1% and 57.2%, respectively, under identical conditions.

### Leakage current indicators trend under different P_u_/P_L_

**Figure 13:**
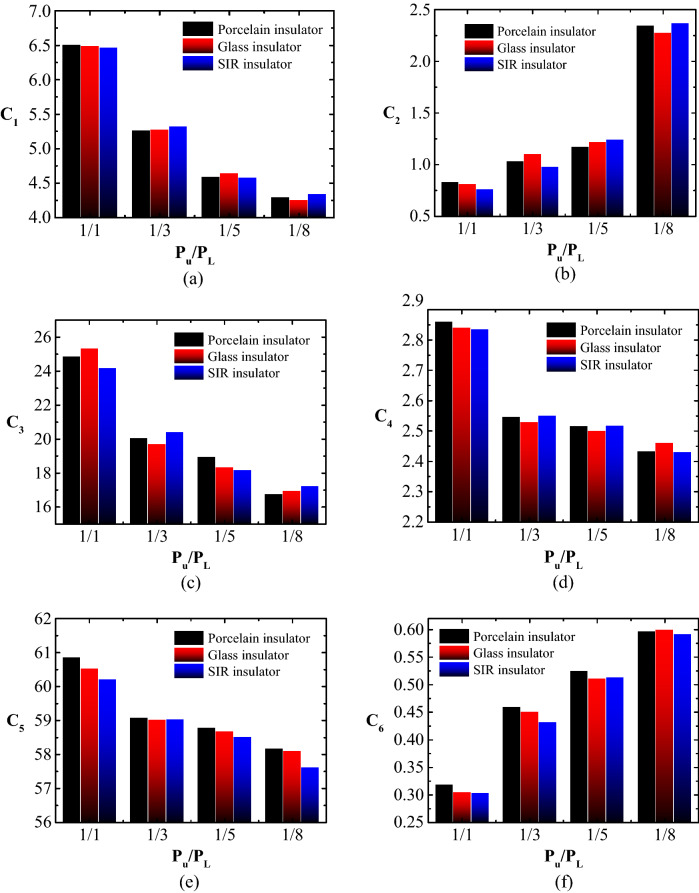
LC indicators versus P_u_/P_L_ for tested insulators under SDD = 0.2 mg/cm^2^, NSDD = 0.35 mg/cm^2^ and Wt = 9 l/h: (**a**) C_1_; (**b**) C_2_; (**c**) C_3_; (**d**) C_4_; (**e**) C_5_; (**f**) C_6_.

Figure 13 shows an example of the relationship between the suggested C_1_-C_6_ indices and P_u_/P_L_ for a dirty porcelain insulator with SDD of 0.2 mg/cm^2^, NSDD of 0.35 mg/cm^2^, and Wt of 9 l/h. It can be noted that rising P_u_/P_L_ leads to increased C_2_ and C_6_ but decreased C_1_, C_3_, C_4_, and C_5_. This suggests that insulators under uniform pollution conditions have a higher probability of flashover occurrence than insulators under non-uniform pollution conditions.

### Flashover voltage indicator results

**Figure 14:**
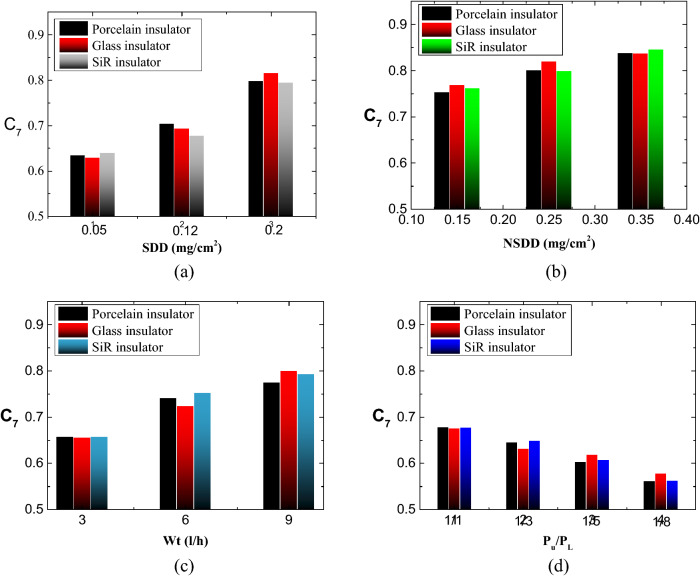
Flashover voltage indicator under different: (**a**) SDD; (**b**) NSDD; (**c**) Wt; (**d**) P_u_/P_L_.

Figure 14 shows an example of the flashover voltage indicator results for porcelain insulator under different levels of SDD, NSDD, Wt and P_u_/P_L_. The range of flashover voltage is between 0 and 1. The SDD and Wt have a significant effect in value of flashover voltage index compared to the NSDD and P_u_/P_L_. The flashover voltage indicator shows that the insulator is in normal condition if its value is less than 0.62, according to the testing results. A flashover voltage indication reading of 0.62 to 0.73 shows that the insulator is in an abnormal state. When the flashover voltage indicator value exceeds 0.73, the insulator is in critical condition.

### Ranges of indices based on test data

The ranges of the proposed indicators for three insulators (porcelain, glass, and SIR insulators) corresponding to the levels of SDD, NSDD, Wt, and P_u_/P_L_ were categorized in this section based on the LC data. The experimental findings confirmed that C_1_, C_3_, C_4_, C_5_ and C_7_ levels rose in proportion to increasing SDD, NSDD, and Wt, but decreasing P_u_/P_L_. Meanwhile, with a rise in SDD, NSDD, and Wt, but a drop in P_u_/P_L_, C_2_ and C_6_ declined. Table 6 displays the prediction of insulators conditions ranges based on empirically derived indicators values.

**Table 6:** Insulator condition dependent on experimentally determined indicators values.

Indicator	Normal range	Abnormal range	Critical range	Pre-flashover value
C_1_	< 1.165	> 1.165and < 2.86	> 2.86	> 10
C_2_	> 59.3	> 18.7 and < 59.3	< 18.7	≈ 0
C_3_	< 1.53	> 1.53 and < 5.1	> 5.1	> 28
C_4_	< 1.6	> 1.6 and < 2	> 2	> 2.8
C_5_	< 15	> 15 and < 45	> 45	> 65
C_6_	> 3	> 1 and < 3	< 1	< 0.4
C_7_	< 0.62	> 0.62 and < 0.73	> 0.73	> 0.85

To understand the prediction process of insulator conditions based on the proposed C_1_-C_7_ indices, the comparison between the indices values in Table 5 and the boundary condition in Table 6 was performed, as follows:

1. The proposed indices values at the normal range were observed in the clean and light pollution cases with Wt less than 4 l/h and NSDD lower than 0.2 mg/cm^2^. In this case, the possibility of discharge occurrence is almost non-existent.
2. According to Table 5, the insulator was in abnormal condition under light pollution (0.05 mg/cm^2^) with heavy wetting Wt (9 l/h) and moderate and heavy levels of NSDD (0.25 and 0.35 mg/cm^2^) for all contamination distribution (P_u_/P_L_) except the 1/8 level. In addition, the insulator under examination displayed an abnormal condition in the presence of moderate pollution (0.12 mg/cm^2^) under moderate wetting Wt (6 l/h), NSDD (0.25 mg/cm^2^), and P_u_/P_L_ (1/1 to 1/5). In this condition, the discharge occurring probability is low, except in cases of extreme wetting, where the possibility of flashover increased.
3. The critical condition of the insulator under test was found under two conditions: first, in medium contamination condition under Wt (9 l/h), NSDD (0.35 mg/cm^2^), and all contamination distribution cases; second, in heavy pollution condition under medium and heavy levels for Wt, NSDD and all pollution distribution cases. The discharge occurring probability in these conditions is high, especially under heavy wet and heavy NSDD.
4. The insulator must be inspected or cleaned when the values of C_1_, C_3_, C_4_, C_5_, and C_7_ indicators are greater than 2.8, 5.1, 2, 45, and 0.73, respectively, and the values for C_2_ and C_6_ indicators are less than 18.7 and 1, respectively.
5. The indicator values at pre-flashover show that these indicators can also be used to detect the flashover phenomenon for contaminated insulators in operation.

### Determination of indices performance

**Figure 15:**
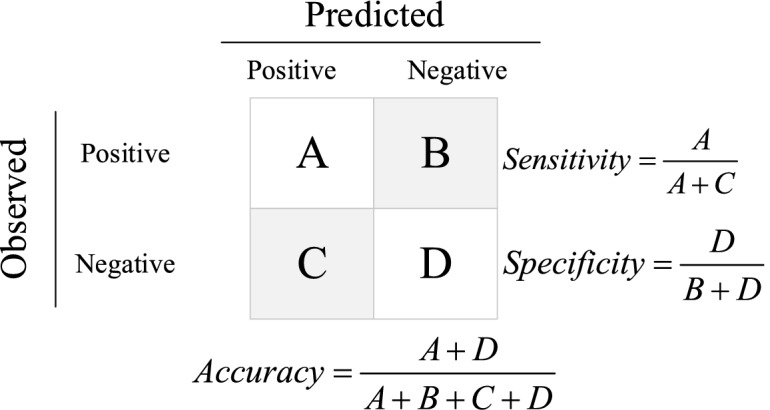
Confusion matrix for determining indices' sensitivity, specificity, and accuracy.

To determine the performance of the proposed indicators precisely, the ability of the indicators to accurately estimate the insulators' state based on 912 tests for three insulators under different conditions was examined. The sensitivity, precision, and accuracy of these indices were calculated using the confusion matrix shown in Fig. 15 during the assessment. The parameters of the confusion matrix were specified based on the insulator condition preparation. The decision of test results and proposed indices are defined as:

A. The tested insulator condition and the indicator prediction for the insulator condition are positive.
B. The tested insulator condition is positive, but the indicator prediction for the insulator condition is negative.
C. The tested insulator condition is negative, but the indicator prediction for the insulator condition is positive.
D. The tested insulator condition and the indicator prediction for the insulator condition are negative.

**Figure 16:**
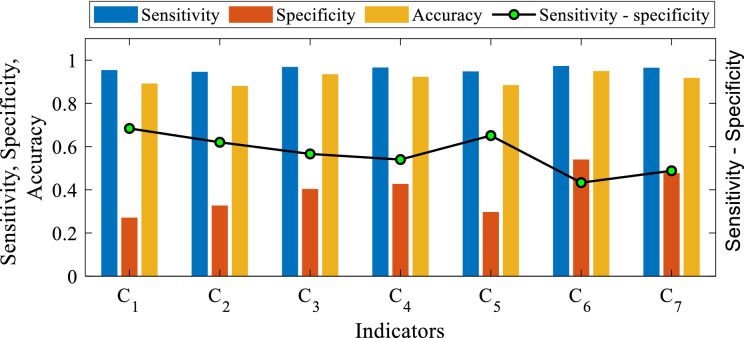
The indicators sensitivity, specificity, accuracy, and the difference between sensitivity and specificity.

Table 7 indicates the positive and negative values for each indicator out of the 912 test samples. The reason for the incorrect predicting findings is either due to a lack of correct application of contaminants on the surface of the insulator or the incorrect indicator diagnosis. The number of prediction results for each indication varied when compared to the total number of test outcomes. The sensitivity, precision, and accuracy results of the proposed indices are shown in Fig. 16.

**Table 7:** The indices' sensitivity, specificity, and accuracy for the 936 tests.

Indicator	A	B	C	D	Sensitivity	Specificity	Accuracy
C_1_	812	62	39	23	0.954	0.271	0.892
C_2_	793	66	45	32	0.946	0.327	0.881
C_3_	852	34	27	23	0.969	0.404	0.935
C_4_	832	43	29	32	0.966	0.427	0.923
C_5_	801	64	44	27	0.948	0.297	0.885
C_6_	862	23	24	27	0.973	0.540	0.950
C_7_	851	35	26	24	0.965	0.477	0.918

The difference in the number of expected outcomes may help identify which indicators are the best, with the indices with the most correct anticipated outcomes being the best. In other words, the indices with the greatest number of correct predicted results will be the most accurate.

According to the indices prediction results, C_6_ (862) had the highest number of right projected outcomes, followed by C_3_ (852), C_7_ (851), C_4_ (832), C_1_ (812), C_5_ (801) and C_2_ (793). Consequently, C_6_ has the best indication accuracy, followed by C_3_, C_4_, C_1_, C_5_, and C_2_ (see Table 7). Meanwhile, C_6_ has also the highest sensitivity, followed by C_7_, C_4_, C_3_, C_2_, C_1_, and C_5_ (see Table 7). Furthermore, the "best" performance is defined as having the smallest fluctuation between sensitivity and specificity^42^. According to Fig. 16, C6 has the least difference between sensitivity and specificity, followed by C_7_, C_4_, C_3_, C_2_, C_5_, and C_1_.

### Leakage current measurement in the practical electric grid

It should be noted that testing the insulators in actual sites differs from typical experimental test setups. Recent studies^43–45^ show that synthetic accelerated tests are used in experimental settings to study flashover states of contaminated insulators. It should be highlighted that all these studies are based on the standard process for insulator tests^35^. IEC standard^35^ describes, for example, test types and methods of pre-contamination procedures in an experiment conducted to imitate real on-site pollution situations. Following the standard in the artificial contamination processes should therefore provide test circumstances similar to real on-site contaminated situations, albeit that some variations and inaccuracies between synthetic pollution test conditions and real on-site circumstances (such as noise in voltage and LC signal) are inevitable.

**Figure 17:**
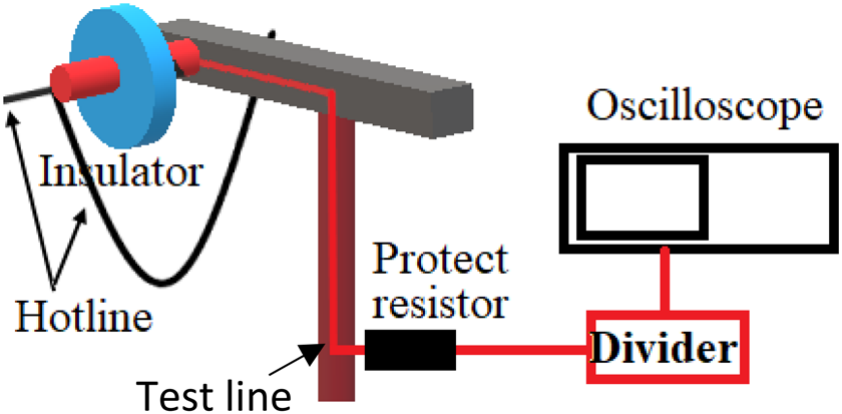
Illustration of on-site insulator testing.

It is noteworthy that the proposed indicators have yet to be tested in real power distribution lines due to the difficulties in accessing real power distribution lines locally. However, effort is currently underway to discuss such a possibility with the local power network provider in accessing a real power distribution line for the purpose of testing the proposed indicators under real operating conditions. Nevertheless, we illustrate in Fig. 17 on how the proposed LC indicators can be used at an on-site power distribution line to predict the condition of contaminated insulators. The ground electrode of the glass insulator on a 11 kV distribution line can be connected to a resistive divider and subsequently to an oscilloscope for signal reading.

## Conclusions

To effectively analyse the stability of contaminated insulators, this article conducted an experimental analysis of leakage current indices. The contamination, wetting rate, non-soluble deposit density, and non-uniform distribution pollution were all studied and executed on the porcelain, glass, and SIR insulators. Then, based on the laboratory test results, the time and frequency characteristics of the leakage current and flashover voltage were extracted and employed as assessment indicators for the insulators' physical conditions. The ranges of the leakage current indicators for four different insulators' conditions were classified. The confusion matrix technique was used for assessing the performance of the proposed indicator. The indicators are all relevant in detecting the condition of contaminated insulators. The C_6_, C_3_, and C_4_ indicators perform better than other indicators in terms of accuracy, with 0.950, 0.935, and 0.923, respectively, according to the confusion matrix analysis. Future research directions linked to the diagnosis of the insulator condition can include, but are not limited to, the following work: (1) testing the proposed indicators in real power distribution lines; (2) designing a device capable of directly detecting the state of the insulator and wirelessly transferring the data based the proposed indicators in this work; and (3) developing a new optimization technique to denoise and classify the signal captured directly from the transmission line based on the insulator's conditions.

## Supplementary Information

Supplementary Information.

## Data Availability

All data generated or analyzed during this study were included at https://github.com/Salem1985-dot/ali.git. All data in this work has been obtained from the insulators test in a high voltage laboratory, Universiti Teknologi Malaysia (UTM), Johor Bahru, Malaysia.
