# Supplementary material for: Leakage current characteristics in estimating insulator reliability: experimental investigation and analysis
Source: Sci Rep. 2022 Sep 2;12:14974. doi: 10.1038/s41598-022-17792-x (PMC9440045; doi:10.1038/s41598-022-17792-x)
Supplement: Supplementary file 1 — Supplementary Information. [file 41598_2022_17792_MOESM1_ESM.docx]

**Appendix A**

**Table A1.** Leakage current components for different pollution levels under 1/3 non-uniform pollution.

| **Insulator type** | | | **Porcelain** | | | | | | | **Glass** | | | | | | | **SIR** | | | | | | |
| --- | --- | --- | --- | --- | --- | --- | --- | --- | --- | --- | --- | --- | --- | --- | --- | --- | --- | --- | --- | --- | --- | --- | --- |
| **SDD** | **NSDD** | **W_t_** | ***I_m_*** | **3rd** | **5th** | **7th** | **9th** | **THD** | **ϕ** | ***I_m_*** | **3rd** | **5th** | **7th** | **9th** | **THD** | **ϕ** | ***I_m_*** | **3rd** | **5th** | **7th** | **9th** | **THD** | **ϕ** |
| 0.05 | 0.15 | 0 | 0.422 | 0.007 | 0.035 | 0.007 | 0.003 | 7.602 | 87.75 | 0.405 | 0.007 | 0.034 | 0.007 | 0.003 | 7.289 | 84.132 | 0.386 | 0.006 | 0.032 | 0.006 | 0.003 | 6.955 | 80.284 |
|  |  | 3 | 0.727 | 0.067 | 0.156 | 0.048 | 0.024 | 9.472 | 81.689 | 0.697 | 0.064 | 0.150 | 0.046 | 0.023 | 9.081 | 78.321 | 0.665 | 0.061 | 0.143 | 0.044 | 0.022 | 8.666 | 74.738 |
|  |  | 6 | 0.962 | 0.087 | 0.173 | 0.072 | 0.032 | 9.528 | 69.525 | 0.922 | 0.083 | 0.166 | 0.069 | 0.031 | 9.135 | 66.659 | 0.880 | 0.080 | 0.158 | 0.066 | 0.029 | 8.717 | 63.609 |
|  |  | 9 | 1.269 | 0.105 | 0.19 | 0.084 | 0.045 | 9.648 | 59.338 | 1.217 | 0.101 | 0.182 | 0.081 | 0.043 | 9.250 | 56.892 | 1.161 | 0.096 | 0.174 | 0.077 | 0.041 | 8.827 | 54.289 |
|  | 0.25 | 0 | 0.6 | 0.011 | 0.066 | 0.007 | 0.006 | 7.31 | 83.173 | 0.575 | 0.011 | 0.063 | 0.007 | 0.006 | 7.009 | 79.744 | 0.549 | 0.010 | 0.060 | 0.006 | 0.005 | 6.688 | 76.096 |
|  |  | 3 | 1.01 | 0.123 | 0.211 | 0.1 | 0.036 | 8.944 | 68.196 | 0.968 | 0.118 | 0.202 | 0.096 | 0.035 | 8.575 | 65.384 | 0.924 | 0.113 | 0.193 | 0.091 | 0.033 | 8.183 | 62.393 |
|  |  | 6 | 1.224 | 0.172 | 0.289 | 0.096 | 0.078 | 8.911 | 55.878 | 1.174 | 0.165 | 0.277 | 0.092 | 0.075 | 8.544 | 53.574 | 1.120 | 0.157 | 0.264 | 0.088 | 0.071 | 8.153 | 51.124 |
|  |  | 9 | 1.511 | 0.181 | 0.311 | 0.106 | 0.068 | 9.059 | 43.94 | 1.449 | 0.174 | 0.298 | 0.102 | 0.065 | 8.686 | 42.128 | 1.382 | 0.166 | 0.285 | 0.097 | 0.062 | 8.288 | 40.201 |
|  | 0.35 | 0 | 0.741 | 0.011 | 0.053 | 0.011 | 0.008 | 7.845 | 80.67 | 0.710 | 0.011 | 0.051 | 0.011 | 0.008 | 7.522 | 77.344 | 0.678 | 0.010 | 0.048 | 0.010 | 0.007 | 7.177 | 73.806 |
|  |  | 3 | 1.328 | 0.161 | 0.217 | 0.088 | 0.046 | 9.237 | 57.896 | 1.273 | 0.154 | 0.208 | 0.084 | 0.044 | 8.856 | 55.509 | 1.215 | 0.147 | 0.199 | 0.081 | 0.042 | 8.451 | 52.970 |
|  |  | 6 | 1.662 | 0.171 | 0.187 | 0.114 | 0.073 | 9.588 | 41.334 | 1.593 | 0.164 | 0.179 | 0.109 | 0.070 | 9.193 | 39.630 | 1.521 | 0.156 | 0.171 | 0.104 | 0.067 | 8.772 | 37.817 |
|  |  | 9 | 2.126 | 0.215 | 0.236 | 0.13 | 0.09 | 10.459 | 34.217 | 2.038 | 0.206 | 0.226 | 0.125 | 0.086 | 10.028 | 32.806 | 1.945 | 0.197 | 0.216 | 0.119 | 0.082 | 9.569 | 31.306 |
| 0.12 | 0.15 | 0 | 0.517 | 0.014 | 0.059 | 0.009 | 0.007 | 6.675 | 73.336 | 0.496 | 0.013 | 0.057 | 0.009 | 0.007 | 6.400 | 70.313 | 0.473 | 0.013 | 0.054 | 0.008 | 0.006 | 6.107 | 67.096 |
|  |  | 3 | 1.203 | 0.268 | 0.251 | 0.033 | 0.065 | 10.024 | 56.866 | 1.153 | 0.257 | 0.241 | 0.032 | 0.062 | 9.611 | 54.522 | 1.101 | 0.245 | 0.230 | 0.030 | 0.059 | 9.171 | 52.027 |
|  |  | 6 | 1.325 | 0.35 | 0.293 | 0.075 | 0.098 | 10.472 | 39.686 | 1.270 | 0.336 | 0.281 | 0.072 | 0.094 | 10.040 | 38.050 | 1.212 | 0.320 | 0.268 | 0.069 | 0.090 | 9.581 | 36.309 |
|  |  | 9 | 1.569 | 0.374 | 0.382 | 0.089 | 0.081 | 10.772 | 27.975 | 1.504 | 0.359 | 0.366 | 0.085 | 0.078 | 10.328 | 26.822 | 1.435 | 0.342 | 0.349 | 0.081 | 0.074 | 9.855 | 25.595 |
|  | 0.25 | 0 | 0.588 | 0.018 | 0.068 | 0.01 | 0.013 | 7.741 | 78.167 | 0.564 | 0.017 | 0.065 | 0.010 | 0.012 | 7.422 | 74.944 | 0.540 | 0.017 | 0.063 | 0.009 | 0.012 | 7.115 | 71.845 |
|  |  | 3 | 1.348 | 0.274 | 0.243 | 0.081 | 0.071 | 9.981 | 54.096 | 1.292 | 0.263 | 0.233 | 0.078 | 0.068 | 9.570 | 51.866 | 1.239 | 0.252 | 0.223 | 0.074 | 0.065 | 9.174 | 49.721 |
|  |  | 6 | 1.536 | 0.352 | 0.29 | 0.094 | 0.077 | 10.381 | 31.127 | 1.473 | 0.337 | 0.278 | 0.090 | 0.074 | 9.953 | 29.844 | 1.412 | 0.324 | 0.267 | 0.086 | 0.071 | 9.541 | 28.609 |
|  |  | 9 | 1.755 | 0.39 | 0.321 | 0.102 | 0.086 | 10.968 | 25.874 | 1.683 | 0.374 | 0.308 | 0.098 | 0.082 | 10.516 | 24.807 | 1.613 | 0.358 | 0.295 | 0.094 | 0.079 | 10.081 | 23.781 |
|  | 0.35 | 0 | 0.759 | 0.028 | 0.071 | 0.025 | 0.019 | 9.176 | 72.543 | 0.728 | 0.027 | 0.068 | 0.024 | 0.018 | 8.798 | 69.552 | 0.698 | 0.026 | 0.065 | 0.023 | 0.017 | 8.434 | 66.676 |
|  |  | 3 | 1.6 | 0.412 | 0.291 | 0.105 | 0.081 | 12.509 | 40.85 | 1.509 | 0.389 | 0.275 | 0.099 | 0.076 | 11.801 | 38.538 | 1.471 | 0.379 | 0.267 | 0.097 | 0.074 | 11.497 | 37.546 |
|  |  | 6 | 1.648 | 0.482 | 0.331 | 0.121 | 0.105 | 13.546 | 21.249 | 1.555 | 0.455 | 0.312 | 0.114 | 0.099 | 12.779 | 20.046 | 1.515 | 0.443 | 0.304 | 0.111 | 0.097 | 12.450 | 19.530 |
|  |  | 9 | 2.109 | 0.495 | 0.339 | 0.174 | 0.081 | 14.852 | 15.399 | 1.990 | 0.467 | 0.320 | 0.164 | 0.076 | 14.011 | 14.527 | 1.938 | 0.455 | 0.312 | 0.160 | 0.074 | 13.651 | 14.153 |
| 0.2 | 0.15 | 0 | 0.68 | 0.04 | 0.08 | 0.04 | 0.03 | 9.19 | 58.545 | 0.642 | 0.038 | 0.075 | 0.038 | 0.028 | 8.670 | 55.231 | 0.625 | 0.037 | 0.074 | 0.037 | 0.028 | 8.447 | 53.810 |
|  |  | 3 | 2.14 | 0.7 | 0.13 | 0.27 | 0.08 | 13.48 | 33.084 | 2.019 | 0.660 | 0.123 | 0.255 | 0.075 | 12.717 | 31.211 | 1.967 | 0.643 | 0.119 | 0.248 | 0.074 | 12.390 | 30.408 |
|  |  | 6 | 2.38 | 0.76 | 0.18 | 0.21 | 0.09 | 14.19 | 18.107 | 2.245 | 0.717 | 0.170 | 0.198 | 0.085 | 13.387 | 17.082 | 2.188 | 0.699 | 0.165 | 0.193 | 0.083 | 13.042 | 16.642 |
|  |  | 9 | 2.95 | 0.79 | 0.19 | 0.18 | 0.11 | 17.38 | 11.907 | 2.783 | 0.745 | 0.179 | 0.170 | 0.104 | 16.396 | 11.233 | 2.711 | 0.726 | 0.175 | 0.165 | 0.101 | 15.974 | 10.944 |
|  | 0.25 | 0 | 0.83 | 0.05 | 0.08 | 0.05 | 0.03 | 9.15 | 43.816 | 0.783 | 0.047 | 0.075 | 0.047 | 0.028 | 8.632 | 41.336 | 0.763 | 0.046 | 0.074 | 0.046 | 0.028 | 8.410 | 40.272 |
|  |  | 3 | 2.37 | 0.69 | 0.21 | 0.17 | 0.11 | 16.32 | 27.244 | 2.236 | 0.651 | 0.198 | 0.160 | 0.104 | 15.396 | 25.702 | 2.178 | 0.634 | 0.193 | 0.156 | 0.101 | 15.000 | 25.040 |
|  |  | 6 | 2.66 | 0.83 | 0.22 | 0.19 | 0.11 | 18.37 | 11.052 | 2.509 | 0.783 | 0.208 | 0.179 | 0.104 | 17.330 | 10.426 | 2.445 | 0.763 | 0.202 | 0.175 | 0.101 | 16.884 | 10.158 |
|  |  | 9 | 3.23 | 0.91 | 0.29 | 0.11 | 0.09 | 20.18 | 1.071 | 3.047 | 0.858 | 0.274 | 0.104 | 0.085 | 19.038 | 1.010 | 2.969 | 0.836 | 0.267 | 0.101 | 0.083 | 18.548 | 0.984 |
|  | 0.35 | 0 | 0.87 | 0.05 | 0.08 | 0.04 | 0.04 | 10.77 | 33.825 | 0.821 | 0.047 | 0.075 | 0.038 | 0.038 | 10.160 | 31.910 | 0.800 | 0.046 | 0.074 | 0.037 | 0.037 | 9.899 | 31.089 |
|  |  | 3 | 3.05 | 0.94 | 0.28 | 0.13 | 0.12 | 20.99 | 4.038 | 2.877 | 0.887 | 0.264 | 0.123 | 0.113 | 19.802 | 3.809 | 2.803 | 0.864 | 0.257 | 0.119 | 0.110 | 19.292 | 3.711 |
|  |  | 6 | 4.62 | 1.07 | 0.36 | 0.25 | 0.1 | 22.49 | 2.132 | 4.358 | 1.009 | 0.340 | 0.236 | 0.094 | 21.217 | 2.011 | 4.246 | 0.983 | 0.331 | 0.230 | 0.092 | 20.671 | 1.960 |
|  |  | 9 | 5.27 | 1.45 | 0.18 | 0.27 | 0.22 | 23.94 | 0.302 | 5.27 | 6.27 | 7.27 | 8.27 | 9.27 | 10.27 | 11.27 | 4.844 | 1.333 | 0.165 | 0.248 | 0.202 | 22.004 | 0.278 |

**Table A2.** Leakage current components for different pollution levels under 1/5 non-uniform pollution.

| **Insulator type** | | | **Porcelain** | | | | | | | **Glass** | | | | | | | **SIR** | | | | | | |
| --- | --- | --- | --- | --- | --- | --- | --- | --- | --- | --- | --- | --- | --- | --- | --- | --- | --- | --- | --- | --- | --- | --- | --- |
| **SDD** | **NSDD** | **W_t_** | ***I_m_*** | **3^rd^** | **5^th^** | **7^th^** | **9^th^** | **THD** | ***ϕ*** | ***I_m_*** | **3^rd^** | **5^th^** | **7^th^** | **9^th^** | **THD** | ***ϕ*** | ***I_m_*** | **3^rd^** | **5^th^** | **7^th^** | **9^th^** | **THD** | ***ϕ*** |
| 0.05 | 0.15 | 0 | 0.368 | 0.005 | 0.036 | 0.007 | 0.003 | 7.330 | 86.359 | 0.353 | 0.005 | 0.035 | 0.007 | 0.003 | 7.028 | 82.799 | 0.337 | 0.005 | 0.033 | 0.006 | 0.003 | 6.706 | 79.011 |
|  |  | 3 | 0.634 | 0.062 | 0.149 | 0.046 | 0.023 | 9.134 | 83.323 | 0.608 | 0.059 | 0.143 | 0.044 | 0.022 | 8.757 | 79.888 | 0.580 | 0.057 | 0.136 | 0.042 | 0.021 | 8.357 | 76.233 |
|  |  | 6 | 0.838 | 0.079 | 0.165 | 0.068 | 0.031 | 9.188 | 70.916 | 0.803 | 0.076 | 0.158 | 0.065 | 0.030 | 8.809 | 67.992 | 0.767 | 0.072 | 0.151 | 0.062 | 0.028 | 8.406 | 64.882 |
|  |  | 9 | 1.106 | 0.099 | 0.181 | 0.080 | 0.043 | 9.304 | 60.525 | 1.060 | 0.095 | 0.174 | 0.077 | 0.041 | 8.920 | 58.030 | 1.012 | 0.091 | 0.166 | 0.073 | 0.039 | 8.512 | 55.375 |
|  | 0.25 | 0 | 0.523 | 0.010 | 0.063 | 0.007 | 0.005 | 7.947 | 84.836 | 0.501 | 0.010 | 0.060 | 0.007 | 0.005 | 7.619 | 81.338 | 0.478 | 0.009 | 0.058 | 0.006 | 0.005 | 7.271 | 77.618 |
|  |  | 3 | 0.881 | 0.098 | 0.190 | 0.066 | 0.034 | 9.723 | 69.560 | 0.845 | 0.094 | 0.182 | 0.063 | 0.033 | 9.322 | 66.692 | 0.806 | 0.090 | 0.174 | 0.060 | 0.031 | 8.896 | 63.641 |
|  |  | 6 | 1.067 | 0.110 | 0.180 | 0.072 | 0.056 | 9.688 | 56.995 | 1.023 | 0.105 | 0.173 | 0.069 | 0.054 | 9.289 | 54.645 | 0.976 | 0.101 | 0.165 | 0.066 | 0.051 | 8.864 | 52.145 |
|  |  | 9 | 1.318 | 0.122 | 0.188 | 0.091 | 0.064 | 9.848 | 44.819 | 1.264 | 0.117 | 0.180 | 0.087 | 0.061 | 9.442 | 42.971 | 1.206 | 0.112 | 0.172 | 0.083 | 0.059 | 9.010 | 41.005 |
|  | 0.35 | 0 | 0.646 | 0.009 | 0.050 | 0.010 | 0.008 | 8.599 | 82.283 | 0.619 | 0.009 | 0.048 | 0.010 | 0.008 | 8.244 | 78.891 | 0.591 | 0.008 | 0.046 | 0.009 | 0.007 | 7.867 | 75.282 |
|  |  | 3 | 1.158 | 0.142 | 0.207 | 0.084 | 0.043 | 10.125 | 59.054 | 1.110 | 0.136 | 0.198 | 0.081 | 0.041 | 9.708 | 56.619 | 1.059 | 0.130 | 0.189 | 0.077 | 0.039 | 9.263 | 54.029 |
|  |  | 6 | 1.449 | 0.148 | 0.178 | 0.109 | 0.070 | 10.509 | 42.161 | 1.389 | 0.142 | 0.171 | 0.105 | 0.067 | 10.076 | 40.423 | 1.326 | 0.135 | 0.163 | 0.100 | 0.064 | 9.615 | 38.574 |
|  |  | 9 | 1.854 | 0.158 | 0.153 | 0.124 | 0.085 | 11.464 | 34.901 | 1.778 | 0.151 | 0.147 | 0.119 | 0.081 | 10.991 | 33.462 | 1.696 | 0.145 | 0.140 | 0.113 | 0.078 | 10.489 | 31.931 |
| 0.12 | 0.15 | 0 | 0.451 | 0.011 | 0.056 | 0.009 | 0.006 | 7.330 | 84.803 | 0.432 | 0.011 | 0.054 | 0.009 | 0.006 | 7.028 | 81.307 | 0.413 | 0.010 | 0.051 | 0.008 | 0.005 | 6.706 | 77.587 |
|  |  | 3 | 1.049 | 0.212 | 0.249 | 0.031 | 0.062 | 11.009 | 38.004 | 1.006 | 0.203 | 0.239 | 0.030 | 0.059 | 10.555 | 36.437 | 0.960 | 0.194 | 0.228 | 0.028 | 0.057 | 10.072 | 34.770 |
|  |  | 6 | 1.156 | 0.295 | 0.279 | 0.071 | 0.093 | 11.500 | 30.480 | 1.108 | 0.283 | 0.267 | 0.068 | 0.089 | 11.026 | 29.223 | 1.058 | 0.270 | 0.255 | 0.065 | 0.085 | 10.522 | 27.887 |
|  |  | 9 | 1.368 | 0.356 | 0.364 | 0.104 | 0.077 | 11.830 | 18.534 | 1.312 | 0.341 | 0.349 | 0.100 | 0.074 | 11.342 | 17.770 | 1.252 | 0.326 | 0.333 | 0.095 | 0.070 | 10.823 | 16.957 |
|  | 0.25 | 0 | 0.512 | 0.015 | 0.065 | 0.010 | 0.013 | 8.821 | 79.730 | 0.491 | 0.014 | 0.062 | 0.010 | 0.012 | 8.457 | 76.443 | 0.471 | 0.014 | 0.060 | 0.009 | 0.012 | 8.108 | 73.281 |
|  |  | 3 | 1.175 | 0.242 | 0.231 | 0.078 | 0.067 | 11.375 | 35.178 | 1.127 | 0.232 | 0.221 | 0.075 | 0.064 | 10.906 | 33.728 | 1.080 | 0.222 | 0.212 | 0.072 | 0.062 | 10.455 | 32.333 |
|  |  | 6 | 1.339 | 0.295 | 0.276 | 0.090 | 0.073 | 11.830 | 31.749 | 1.284 | 0.283 | 0.265 | 0.086 | 0.070 | 11.342 | 30.440 | 1.231 | 0.271 | 0.254 | 0.083 | 0.067 | 10.873 | 29.181 |
|  |  | 9 | 1.530 | 0.310 | 0.306 | 0.097 | 0.082 | 12.500 | 16.391 | 1.467 | 0.297 | 0.293 | 0.093 | 0.079 | 11.985 | 15.715 | 1.406 | 0.285 | 0.281 | 0.089 | 0.075 | 11.489 | 15.065 |
|  | 0.35 | 0 | 0.662 | 0.023 | 0.068 | 0.024 | 0.018 | 8.848 | 73.994 | 0.635 | 0.022 | 0.065 | 0.023 | 0.017 | 8.483 | 70.943 | 0.608 | 0.021 | 0.063 | 0.022 | 0.017 | 8.132 | 68.009 |
|  |  | 3 | 1.395 | 0.331 | 0.277 | 0.100 | 0.077 | 12.063 | 41.667 | 1.316 | 0.312 | 0.261 | 0.094 | 0.073 | 11.380 | 39.308 | 1.282 | 0.304 | 0.255 | 0.092 | 0.071 | 11.087 | 38.297 |
|  |  | 6 | 1.437 | 0.414 | 0.316 | 0.115 | 0.100 | 13.063 | 21.674 | 1.356 | 0.391 | 0.298 | 0.108 | 0.094 | 12.324 | 20.447 | 1.321 | 0.381 | 0.290 | 0.106 | 0.092 | 12.006 | 19.921 |
|  |  | 9 | 1.839 | 0.457 | 0.323 | 0.161 | 0.077 | 14.321 | 15.706 | 1.735 | 0.431 | 0.305 | 0.152 | 0.073 | 13.510 | 14.817 | 1.690 | 0.420 | 0.297 | 0.148 | 0.071 | 13.163 | 14.436 |
| 0.2 | 0.15 | 0 | 0.574 | 0.035 | 0.074 | 0.035 | 0.025 | 8.857 | 59.716 | 0.542 | 0.033 | 0.070 | 0.033 | 0.024 | 8.356 | 56.336 | 0.528 | 0.032 | 0.068 | 0.032 | 0.023 | 8.141 | 54.886 |
|  |  | 3 | 1.862 | 0.662 | 0.216 | 0.154 | 0.131 | 13.000 | 33.745 | 1.757 | 0.625 | 0.204 | 0.145 | 0.124 | 12.264 | 31.835 | 1.711 | 0.608 | 0.199 | 0.142 | 0.120 | 11.949 | 31.016 |
|  |  | 6 | 2.074 | 0.724 | 0.250 | 0.139 | 0.085 | 13.679 | 18.470 | 1.957 | 0.683 | 0.236 | 0.131 | 0.080 | 12.905 | 17.425 | 1.906 | 0.665 | 0.230 | 0.128 | 0.078 | 12.573 | 16.976 |
|  |  | 9 | 2.575 | 0.718 | 0.177 | 0.130 | 0.100 | 31.045 | 12.145 | 2.429 | 0.677 | 0.167 | 0.123 | 0.094 | 29.288 | 11.458 | 2.367 | 0.660 | 0.163 | 0.119 | 0.092 | 28.534 | 11.163 |
|  | 0.25 | 0 | 0.727 | 0.042 | 0.077 | 0.049 | 0.025 | 8.821 | 44.693 | 0.686 | 0.040 | 0.073 | 0.046 | 0.024 | 8.322 | 42.163 | 0.668 | 0.039 | 0.071 | 0.045 | 0.023 | 8.108 | 41.078 |
|  |  | 3 | 2.067 | 0.655 | 0.209 | 0.162 | 0.100 | 15.741 | 27.788 | 1.950 | 0.618 | 0.197 | 0.153 | 0.094 | 14.850 | 26.215 | 1.900 | 0.602 | 0.192 | 0.149 | 0.092 | 14.468 | 25.540 |
|  |  | 6 | 2.321 | 0.693 | 0.208 | 0.177 | 0.108 | 17.714 | 11.273 | 2.190 | 0.654 | 0.196 | 0.167 | 0.102 | 16.711 | 10.635 | 2.133 | 0.637 | 0.191 | 0.163 | 0.099 | 16.281 | 10.361 |
|  |  | 9 | 2.814 | 0.870 | 0.217 | 0.200 | 0.085 | 19.455 | 1.093 | 2.655 | 0.821 | 0.205 | 0.189 | 0.080 | 18.354 | 1.031 | 2.586 | 0.800 | 0.199 | 0.184 | 0.078 | 17.881 | 1.005 |
|  | 0.35 | 0 | 0.755 | 0.044 | 0.079 | 0.037 | 0.039 | 10.384 | 34.502 | 0.712 | 0.042 | 0.075 | 0.035 | 0.037 | 9.796 | 32.549 | 0.694 | 0.040 | 0.073 | 0.034 | 0.036 | 9.544 | 31.711 |
|  |  | 3 | 2.792 | 0.709 | 0.170 | 0.169 | 0.116 | 20.241 | 4.118 | 2.634 | 0.669 | 0.160 | 0.159 | 0.109 | 19.095 | 3.885 | 2.566 | 0.652 | 0.156 | 0.155 | 0.107 | 18.604 | 3.785 |
|  |  | 6 | 3.285 | 1.017 | 0.247 | 0.239 | 0.092 | 21.688 | 2.175 | 3.099 | 0.959 | 0.233 | 0.225 | 0.087 | 20.460 | 2.052 | 3.019 | 0.935 | 0.227 | 0.220 | 0.085 | 19.934 | 1.999 |
|  |  | 9 | 4.592 | 1.379 | 0.262 | 0.254 | 0.208 | 23.089 | 0.044 | 1.239 | 0.325 | 0.229 | 0.187 | 20.125 | 0.495 | 5.067 | 1.192 | 0.313 | 0.220 | 0.179 | 19.351 | 0.476 | 1.239 |

**Table A3.** Leakage current components for different pollution levels under 1/8 non-uniform pollution.

| **Insulator type** | | | **Porcelain** | | | | | | | **Glass** | | | | | | | **SIR** | | | | | | |
| --- | --- | --- | --- | --- | --- | --- | --- | --- | --- | --- | --- | --- | --- | --- | --- | --- | --- | --- | --- | --- | --- | --- | --- |
| **SDD** | **NSDD** | **W_t_** | ***I_m_*** | **3^rd^** | **5^th^** | **7^th^** | **9^th^** | **THD** | ***ϕ*** | ***I_m_*** | **3^rd^** | **5^th^** | **7^th^** | **9^th^** | **THD** | ***ϕ*** | ***I_m_*** | **3^rd^** | **5^th^** | **7^th^** | **9^th^** | **THD** | ***ϕ*** |
| 0.05 | 0.15 | 0 | 0.344 | 0.005 | 0.034 | 0.006 | 0.003 | 6.65 | 86.618 | 0.330 | 0.005 | 0.033 | 0.006 | 0.003 | 6.376 | 83.047 | 0.315 | 0.005 | 0.031 | 0.005 | 0.003 | 6.084 | 79.248 |
|  |  | 3 | 0.593 | 0.055 | 0.139 | 0.043 | 0.022 | 8.28 | 83.573 | 0.569 | 0.053 | 0.133 | 0.041 | 0.021 | 7.939 | 80.128 | 0.543 | 0.050 | 0.127 | 0.039 | 0.020 | 7.575 | 76.462 |
|  |  | 6 | 0.784 | 0.070 | 0.154 | 0.064 | 0.029 | 8.33 | 71.128 | 0.752 | 0.067 | 0.148 | 0.061 | 0.028 | 7.987 | 68.196 | 0.717 | 0.064 | 0.141 | 0.059 | 0.027 | 7.621 | 65.076 |
|  |  | 9 | 1.034 | 0.082 | 0.169 | 0.075 | 0.040 | 8.43 | 60.707 | 0.991 | 0.079 | 0.162 | 0.072 | 0.038 | 8.082 | 58.204 | 0.946 | 0.075 | 0.155 | 0.069 | 0.037 | 7.713 | 55.542 |
|  | 0.25 | 0 | 0.489 | 0.009 | 0.062 | 0.006 | 0.005 | 7.21 | 85.090 | 0.469 | 0.009 | 0.059 | 0.006 | 0.005 | 6.913 | 81.582 | 0.447 | 0.008 | 0.057 | 0.005 | 0.005 | 6.597 | 77.850 |
|  |  | 3 | 0.823 | 0.088 | 0.177 | 0.062 | 0.032 | 8.82 | 69.769 | 0.789 | 0.084 | 0.170 | 0.059 | 0.031 | 8.456 | 66.893 | 0.753 | 0.081 | 0.162 | 0.057 | 0.029 | 8.070 | 63.833 |
|  |  | 6 | 0.997 | 0.095 | 0.168 | 0.068 | 0.052 | 8.78 | 57.166 | 0.956 | 0.091 | 0.161 | 0.065 | 0.050 | 8.418 | 54.809 | 0.912 | 0.087 | 0.154 | 0.062 | 0.048 | 8.033 | 52.302 |
|  |  | 9 | 1.232 | 0.114 | 0.175 | 0.085 | 0.060 | 8.93 | 44.953 | 1.181 | 0.109 | 0.168 | 0.081 | 0.058 | 8.562 | 43.100 | 1.127 | 0.104 | 0.160 | 0.078 | 0.055 | 8.170 | 41.128 |
|  | 0.35 | 0 | 0.604 | 0.009 | 0.055 | 0.009 | 0.007 | 7.80 | 82.530 | 0.579 | 0.009 | 0.053 | 0.009 | 0.007 | 7.478 | 79.128 | 0.553 | 0.008 | 0.050 | 0.008 | 0.006 | 7.136 | 75.508 |
|  |  | 3 | 1.082 | 0.130 | 0.193 | 0.089 | 0.041 | 9.18 | 59.231 | 1.037 | 0.125 | 0.185 | 0.085 | 0.039 | 8.802 | 56.789 | 0.990 | 0.119 | 0.177 | 0.081 | 0.038 | 8.399 | 54.191 |
|  |  | 6 | 1.354 | 0.132 | 0.167 | 0.102 | 0.065 | 9.53 | 42.287 | 1.298 | 0.127 | 0.160 | 0.098 | 0.062 | 9.137 | 40.544 | 1.239 | 0.121 | 0.153 | 0.093 | 0.059 | 8.719 | 38.689 |
|  |  | 9 | 1.733 | 0.239 | 0.377 | 0.116 | 0.080 | 10.39 | 35.006 | 1.662 | 0.229 | 0.361 | 0.111 | 0.077 | 9.962 | 33.563 | 1.586 | 0.219 | 0.345 | 0.106 | 0.073 | 9.506 | 32.027 |
| 0.12 | 0.15 | 0 | 0.421 | 0.010 | 0.067 | 0.010 | 0.006 | 6.65 | 75.027 | 0.404 | 0.010 | 0.064 | 0.010 | 0.006 | 6.376 | 71.934 | 0.385 | 0.009 | 0.061 | 0.009 | 0.005 | 6.084 | 68.643 |
|  |  | 3 | 0.981 | 0.159 | 0.224 | 0.029 | 0.058 | 9.98 | 58.178 | 0.941 | 0.152 | 0.215 | 0.028 | 0.056 | 9.569 | 55.779 | 0.898 | 0.145 | 0.205 | 0.027 | 0.053 | 9.131 | 53.228 |
|  |  | 6 | 1.080 | 0.247 | 0.261 | 0.067 | 0.087 | 10.43 | 40.601 | 1.035 | 0.237 | 0.250 | 0.064 | 0.083 | 10.000 | 38.927 | 0.988 | 0.226 | 0.239 | 0.061 | 0.080 | 9.543 | 37.146 |
|  |  | 9 | 1.279 | 0.287 | 0.340 | 0.079 | 0.072 | 10.73 | 28.620 | 1.226 | 0.275 | 0.326 | 0.076 | 0.069 | 10.288 | 27.440 | 1.170 | 0.263 | 0.311 | 0.072 | 0.066 | 9.817 | 26.185 |
|  | 0.25 | 0 | 0.479 | 0.015 | 0.090 | 0.009 | 0.012 | 8.00 | 79.969 | 0.459 | 0.014 | 0.086 | 0.009 | 0.012 | 7.670 | 76.672 | 0.440 | 0.014 | 0.083 | 0.008 | 0.011 | 7.353 | 73.501 |
|  |  | 3 | 1.098 | 0.214 | 0.216 | 0.073 | 0.063 | 10.31 | 55.343 | 1.053 | 0.205 | 0.207 | 0.070 | 0.060 | 9.885 | 53.061 | 1.009 | 0.197 | 0.199 | 0.067 | 0.058 | 9.476 | 50.867 |
|  |  | 6 | 1.252 | 0.264 | 0.258 | 0.084 | 0.068 | 10.73 | 31.844 | 1.200 | 0.253 | 0.247 | 0.081 | 0.065 | 10.288 | 30.531 | 1.151 | 0.243 | 0.237 | 0.077 | 0.063 | 9.862 | 29.268 |
|  |  | 9 | 1.430 | 0.284 | 0.286 | 0.091 | 0.077 | 11.33 | 26.470 | 1.371 | 0.272 | 0.274 | 0.087 | 0.074 | 10.863 | 25.379 | 1.314 | 0.261 | 0.263 | 0.084 | 0.071 | 10.414 | 24.329 |
|  | 0.35 | 0 | 0.619 | 0.017 | 0.063 | 0.022 | 0.017 | 8.02 | 74.216 | 0.593 | 0.016 | 0.060 | 0.021 | 0.016 | 7.689 | 71.156 | 0.569 | 0.016 | 0.058 | 0.020 | 0.016 | 7.371 | 68.213 |
|  |  | 3 | 1.304 | 0.293 | 0.259 | 0.093 | 0.072 | 10.94 | 41.792 | 1.230 | 0.276 | 0.244 | 0.088 | 0.068 | 10.321 | 39.426 | 1.199 | 0.269 | 0.238 | 0.085 | 0.066 | 10.055 | 38.412 |
|  |  | 6 | 1.343 | 0.327 | 0.295 | 0.108 | 0.093 | 11.84 | 21.739 | 1.267 | 0.308 | 0.278 | 0.102 | 0.088 | 11.170 | 20.508 | 1.234 | 0.301 | 0.271 | 0.099 | 0.085 | 10.882 | 19.981 |
|  |  | 9 | 1.719 | 0.372 | 0.302 | 0.150 | 0.072 | 12.98 | 15.754 | 1.622 | 0.351 | 0.285 | 0.142 | 0.068 | 12.245 | 14.862 | 1.580 | 0.342 | 0.278 | 0.138 | 0.066 | 11.930 | 14.480 |
| 0.2 | 0.15 | 0 | 0.536 | 0.026 | 0.069 | 0.032 | 0.023 | 8.03 | 59.895 | 0.506 | 0.025 | 0.065 | 0.030 | 0.022 | 7.575 | 56.505 | 0.493 | 0.024 | 0.063 | 0.029 | 0.021 | 7.381 | 55.051 |
|  |  | 3 | 1.740 | 0.619 | 0.202 | 0.238 | 0.122 | 11.79 | 33.847 | 1.642 | 0.584 | 0.191 | 0.225 | 0.115 | 11.123 | 31.931 | 1.599 | 0.569 | 0.186 | 0.219 | 0.112 | 10.836 | 31.109 |
|  |  | 6 | 1.938 | 0.677 | 0.266 | 0.187 | 0.079 | 12.40 | 18.525 | 1.828 | 0.639 | 0.251 | 0.176 | 0.075 | 11.698 | 17.476 | 1.781 | 0.622 | 0.244 | 0.172 | 0.073 | 11.397 | 17.027 |
|  |  | 9 | 2.406 | 0.626 | 0.166 | 0.194 | 0.094 | 28.15 | 12.181 | 2.270 | 0.591 | 0.157 | 0.183 | 0.089 | 26.557 | 11.492 | 2.211 | 0.575 | 0.153 | 0.178 | 0.086 | 25.873 | 11.196 |
|  | 0.25 | 0 | 0.679 | 0.033 | 0.072 | 0.045 | 0.023 | 8.00 | 44.827 | 0.641 | 0.031 | 0.068 | 0.042 | 0.022 | 7.547 | 42.290 | 0.624 | 0.030 | 0.066 | 0.041 | 0.021 | 7.353 | 41.201 |
|  |  | 3 | 1.932 | 0.612 | 0.223 | 0.151 | 0.094 | 14.27 | 27.872 | 1.823 | 0.577 | 0.210 | 0.142 | 0.089 | 13.462 | 26.294 | 1.776 | 0.563 | 0.205 | 0.139 | 0.086 | 13.116 | 25.618 |
|  |  | 6 | 2.169 | 0.648 | 0.194 | 0.166 | 0.101 | 16.06 | 11.307 | 2.046 | 0.611 | 0.183 | 0.157 | 0.095 | 15.151 | 10.667 | 1.994 | 0.596 | 0.178 | 0.153 | 0.093 | 14.761 | 10.392 |
|  |  | 9 | 2.630 | 0.814 | 0.259 | 0.187 | 0.079 | 17.64 | 1.096 | 2.481 | 0.768 | 0.244 | 0.176 | 0.075 | 16.642 | 1.034 | 2.417 | 0.748 | 0.238 | 0.172 | 0.073 | 16.213 | 1.007 |
|  | 0.35 | 0 | 0.705 | 0.036 | 0.073 | 0.035 | 0.036 | 9.41 | 34.605 | 0.665 | 0.034 | 0.069 | 0.033 | 0.034 | 8.877 | 32.646 | 0.648 | 0.033 | 0.067 | 0.032 | 0.033 | 8.649 | 31.806 |
|  |  | 3 | 1.674 | 0.662 | 0.252 | 0.158 | 0.108 | 18.35 | 4.131 | 1.579 | 0.625 | 0.238 | 0.149 | 0.102 | 17.311 | 3.897 | 1.539 | 0.608 | 0.232 | 0.145 | 0.099 | 16.866 | 3.797 |
|  |  | 6 | 2.136 | 0.950 | 0.324 | 0.223 | 0.086 | 19.66 | 2.181 | 2.015 | 0.896 | 0.306 | 0.210 | 0.081 | 18.547 | 2.058 | 1.963 | 0.873 | 0.298 | 0.205 | 0.079 | 18.070 | 2.005 |
|  |  | 9 | 4.292 | 1.289 | 0.338 | 0.238 | 0.194 | 20.93 | 0.515 | 1.216 | 0.319 | 0.225 | 0.183 | 19.745 | 0.486 | 4.972 | 1.147 | 0.301 | 0.212 | 0.173 | 18.628 | 0.458 | 1.216 |

**Appendix B**

**Table B1.** LC indicators under various SDD, wetting rate Wl and NSDD for non-uniform polluted Glass insulator.

| P_1_/P_2_ | | | *1/3* | | | | | | *1/5* | | | | | | *1/8* | | | | | |
| --- | --- | --- | --- | --- | --- | --- | --- | --- | --- | --- | --- | --- | --- | --- | --- | --- | --- | --- | --- | --- |
| SDD  mg/cm^2^ | NSDD  mg/cm^2^ | Wl  l/h | *C_1_* | *C_2_* | *C_3_* | *C_4_* | *C_5_* | *C_6_* | *C_1_* | *C_2_* | *C_3_* | *C_4_* | *C_5_* | *C_6_* | *C_1_* | *C_2_* | *C_3_* | *C_4_* | *C_5_* | *C_6_* |
| 0.05 | 0.15 | 0 | 0.41 | 82.75 | 0.14 | 1.40 | 7.79 | 6.80 | 0.36 | 84.40 | 0.13 | 1.40 | 7.75 | 8.23 | 0.33 | 84.66 | 0.13 | 1.39 | 7.67 | 8.79 |
|  |  | 3 | 0.71 | 79.84 | 0.36 | 1.49 | 9.70 | 3.34 | 0.62 | 81.43 | 0.35 | 1.49 | 9.66 | 3.45 | 0.58 | 81.68 | 0.35 | 1.48 | 9.56 | 3.64 |
|  |  | 6 | 0.94 | 67.95 | 0.76 | 1.52 | 10.71 | 3.11 | 0.82 | 69.31 | 0.75 | 1.51 | 10.66 | 3.27 | 0.76 | 69.52 | 0.75 | 1.51 | 10.55 | 3.43 |
|  |  | 9 | 1.24 | 57.99 | 1.01 | 1.53 | 13.68 | 2.97 | 1.08 | 59.16 | 1.00 | 1.53 | 13.61 | 3.00 | 1.01 | 59.33 | 0.99 | 1.52 | 13.47 | 3.37 |
|  | 0.25 | 0 | 0.59 | 81.28 | 0.29 | 1.54 | 8.44 | 6.68 | 0.51 | 82.92 | 0.29 | 1.54 | 8.41 | 7.70 | 0.48 | 83.16 | 0.29 | 1.53 | 8.32 | 8.03 |
|  |  | 3 | 0.98 | 66.01 | 0.67 | 1.50 | 10.23 | 2.73 | 0.85 | 67.32 | 0.67 | 1.50 | 10.18 | 2.86 | 0.79 | 67.53 | 0.66 | 1.49 | 10.08 | 2.98 |
|  |  | 6 | 1.18 | 54.08 | 1.04 | 1.51 | 12.08 | 2.60 | 1.04 | 55.17 | 1.03 | 1.51 | 12.01 | 2.71 | 0.97 | 55.33 | 1.02 | 1.50 | 11.89 | 2.92 |
|  |  | 9 | 1.46 | 42.53 | 2.99 | 1.53 | 14.12 | 2.58 | 1.28 | 43.38 | 2.97 | 1.52 | 14.05 | 2.74 | 1.19 | 43.51 | 2.94 | 1.52 | 13.91 | 2.74 |
|  | 0.35 | 0 | 0.72 | 78.08 | 0.50 | 1.54 | 9.05 | 6.05 | 0.63 | 79.64 | 0.50 | 1.54 | 9.00 | 7.18 | 0.58 | 79.88 | 0.49 | 1.53 | 8.91 | 8.03 |
|  |  | 3 | 1.29 | 56.04 | 0.84 | 1.51 | 12.53 | 2.11 | 1.12 | 57.15 | 0.84 | 1.50 | 12.48 | 2.28 | 1.05 | 57.33 | 0.83 | 1.50 | 12.34 | 2.40 |
|  |  | 6 | 1.61 | 40.00 | 2.93 | 1.52 | 14.82 | 2.13 | 1.40 | 40.81 | 2.92 | 1.52 | 14.74 | 2.33 | 1.31 | 40.93 | 2.89 | 1.51 | 14.60 | 2.45 |
|  |  | 9 | 2.06 | 33.12 | 5.45 | 1.53 | 17.70 | 2.05 | 1.79 | 33.78 | 5.42 | 1.53 | 17.62 | 2.23 | 1.67 | 33.89 | 5.37 | 1.53 | 17.43 | 2.32 |
| 0.12 | 0.15 | 0 | 0.52 | 73.11 | 0.63 | 1.59 | 7.94 | 5.32 | 0.45 | 74.56 | 0.62 | 1.59 | 7.90 | 6.27 | 0.42 | 74.79 | 0.62 | 1.58 | 7.82 | 7.85 |
|  |  | 3 | 1.20 | 56.69 | 2.24 | 1.53 | 14.83 | 1.30 | 1.05 | 57.81 | 2.23 | 1.53 | 14.76 | 1.61 | 0.98 | 57.99 | 2.21 | 1.52 | 14.60 | 1.94 |
|  |  | 6 | 1.33 | 39.56 | 4.68 | 1.58 | 18.27 | 1.33 | 1.16 | 40.35 | 4.66 | 1.57 | 18.18 | 1.50 | 1.08 | 40.47 | 4.61 | 1.57 | 17.99 | 1.67 |
|  |  | 9 | 1.56 | 27.88 | 8.06 | 2.21 | 22.50 | 1.48 | 1.37 | 28.44 | 8.01 | 2.21 | 22.39 | 1.53 | 1.28 | 28.53 | 7.94 | 2.20 | 22.16 | 1.70 |
|  | 0.25 | 0 | 0.59 | 77.92 | 0.78 | 2.41 | 9.56 | 5.04 | 0.51 | 79.48 | 0.78 | 2.41 | 9.51 | 5.71 | 0.48 | 79.71 | 0.77 | 2.40 | 9.41 | 7.33 |
|  |  | 3 | 1.35 | 53.93 | 5.61 | 1.59 | 21.04 | 1.44 | 1.18 | 55.00 | 5.58 | 1.59 | 20.93 | 1.56 | 1.10 | 55.16 | 5.53 | 1.58 | 20.71 | 1.63 |
|  |  | 6 | 1.54 | 31.03 | 6.73 | 2.16 | 24.43 | 1.31 | 1.34 | 31.65 | 6.69 | 2.16 | 24.31 | 1.48 | 1.25 | 31.74 | 6.63 | 2.15 | 24.06 | 1.55 |
|  |  | 9 | 1.74 | 25.79 | 8.55 | 2.40 | 30.94 | 1.31 | 1.53 | 26.31 | 8.50 | 2.40 | 30.79 | 1.56 | 1.43 | 26.39 | 8.42 | 2.39 | 30.46 | 1.59 |
|  | 0.35 | 0 | 0.76 | 72.31 | 0.79 | 2.57 | 9.59 | 4.15 | 0.66 | 73.75 | 0.79 | 2.57 | 9.54 | 4.69 | 0.62 | 73.98 | 0.78 | 2.56 | 9.44 | 5.88 |
|  |  | 3 | 1.59 | 40.72 | 5.13 | 1.61 | 29.53 | 1.16 | 1.40 | 41.54 | 5.11 | 1.60 | 29.38 | 1.37 | 1.30 | 41.66 | 5.06 | 1.60 | 29.08 | 1.45 |
|  |  | 6 | 1.64 | 21.18 | 7.33 | 2.20 | 33.51 | 1.16 | 1.44 | 21.60 | 7.29 | 2.20 | 33.34 | 1.28 | 1.34 | 21.67 | 7.22 | 2.19 | 32.99 | 1.52 |
|  |  | 9 | 2.10 | 15.35 | 10.32 | 2.47 | 40.69 | 1.20 | 1.83 | 15.66 | 10.26 | 2.47 | 40.48 | 1.23 | 1.71 | 15.70 | 10.17 | 2.46 | 40.06 | 1.41 |
| 0.2 | 0.15 | 0 | 0.69 | 59.32 | 1.04 | 2.68 | 9.76 | 3.47 | 0.58 | 60.51 | 1.03 | 2.67 | 9.71 | 3.83 | 0.55 | 60.69 | 1.02 | 2.66 | 9.60 | 4.78 |
|  |  | 3 | 2.17 | 33.52 | 5.96 | 1.78 | 35.97 | 0.68 | 1.88 | 34.19 | 5.93 | 1.78 | 35.79 | 0.77 | 1.76 | 34.30 | 5.87 | 1.78 | 35.41 | 0.92 |
|  |  | 6 | 2.41 | 18.35 | 7.73 | 2.41 | 40.65 | 0.64 | 2.10 | 18.71 | 7.68 | 2.41 | 40.45 | 0.67 | 1.97 | 18.76 | 7.61 | 2.40 | 40.02 | 0.80 |
|  |  | 9 | 2.99 | 12.07 | 11.42 | 2.54 | 43.06 | 0.61 | 2.60 | 12.30 | 11.36 | 2.54 | 42.84 | 0.58 | 2.44 | 12.34 | 11.25 | 2.53 | 42.39 | 0.73 |
|  | 0.25 | 0 | 0.84 | 44.40 | 1.28 | 2.74 | 10.61 | 3.32 | 0.74 | 45.28 | 1.28 | 2.73 | 10.55 | 3.63 | 0.69 | 45.42 | 1.26 | 2.72 | 10.44 | 4.37 |
|  |  | 3 | 2.40 | 27.60 | 9.69 | 1.84 | 44.88 | 0.72 | 2.10 | 28.16 | 9.64 | 1.83 | 44.66 | 0.73 | 1.96 | 28.24 | 9.54 | 1.83 | 44.19 | 0.77 |
|  |  | 6 | 2.70 | 11.20 | 11.32 | 2.49 | 47.06 | 0.64 | 2.35 | 11.42 | 11.25 | 2.48 | 46.83 | 0.72 | 2.20 | 11.46 | 11.14 | 2.47 | 46.33 | 0.72 |
|  |  | 9 | 3.27 | 1.08 | 15.25 | 2.50 | 52.91 | 0.55 | 2.85 | 1.10 | 15.16 | 2.50 | 52.64 | 0.59 | 2.66 | 1.11 | 15.02 | 2.49 | 52.10 | 0.66 |
|  | 0.35 | 0 | 0.88 | 34.28 | 0.64 | 2.66 | 10.46 | 3.25 | 0.76 | 34.95 | 0.64 | 2.66 | 10.41 | 3.53 | 0.72 | 35.07 | 0.63 | 2.64 | 10.29 | 4.10 |
|  |  | 3 | 3.09 | 4.09 | 10.50 | 1.96 | 50.83 | 0.57 | 2.83 | 4.17 | 10.44 | 1.95 | 50.58 | 0.65 | 1.69 | 4.18 | 10.33 | 1.95 | 50.04 | 0.79 |
|  |  | 6 | 4.68 | 2.16 | 12.94 | 2.52 | 58.32 | 0.68 | 3.33 | 2.20 | 12.87 | 2.52 | 58.03 | 0.58 | 2.17 | 2.21 | 12.75 | 2.51 | 57.43 | 0.68 |
|  |  | 9 | 5.34 | 0.54 | 20.32 | 2.58 | 59.87 | 0.47 | 4.65 | 0.23 | 19.20 | 2.57 | 59.56 | 0.54 | 4.35 | 0.12 | 16.98 | 2.56 | 58.95 | 0.61 |

**Table B2.** LC indicators under various SDD, wetting rate Wt and NSDD for non-uniform polluted SIR insulator.

| P1/P2 | | | *1/3* | | | | | | *1/5* | | | | | | *1/8* | | | | | |
| --- | --- | --- | --- | --- | --- | --- | --- | --- | --- | --- | --- | --- | --- | --- | --- | --- | --- | --- | --- | --- |
| SDD  mg/cm^2^ | NSDD  mg/cm^2^ | W_t_  l/h | *C_1_* | *C_2_* | *C_3_* | *C_4_* | *C_5_* | *C_6_* | *C_1_* | *C_2_* | *C_3_* | *C_4_* | *C_5_* | *C_6_* | *C_1_* | *C_2_* | *C_3_* | *C_4_* | *C_5_* | *C_6_* |
| 0.05 | 0.15 | 0 | 0.43 | 85.27 | 0.14 | 1.44 | 8.03 | 7.01 | 0.37 | 86.97 | 0.13 | 1.44 | 7.99 | 8.48 | 0.34 | 87.23 | 0.13 | 1.43 | 7.91 | 9.05 |
|  |  | 3 | 0.74 | 82.27 | 0.37 | 1.53 | 10.00 | 3.44 | 0.63 | 83.91 | 0.36 | 1.53 | 9.95 | 3.55 | 0.59 | 84.16 | 0.36 | 1.52 | 9.85 | 3.75 |
|  |  | 6 | 0.97 | 70.02 | 0.79 | 1.57 | 11.04 | 3.20 | 0.85 | 71.42 | 0.78 | 1.56 | 10.99 | 3.37 | 0.79 | 71.63 | 0.78 | 1.56 | 10.87 | 3.53 |
|  |  | 9 | 1.28 | 59.76 | 1.04 | 1.58 | 14.10 | 3.06 | 1.12 | 60.96 | 1.03 | 1.58 | 14.03 | 3.09 | 1.04 | 61.14 | 1.02 | 1.57 | 13.88 | 3.47 |
|  | 0.25 | 0 | 0.60 | 83.76 | 0.30 | 1.59 | 8.70 | 6.89 | 0.52 | 85.44 | 0.30 | 1.59 | 8.66 | 7.94 | 0.49 | 85.69 | 0.30 | 1.58 | 8.57 | 8.28 |
|  |  | 3 | 1.02 | 68.68 | 0.69 | 1.56 | 10.64 | 2.84 | 0.89 | 70.05 | 0.69 | 1.56 | 10.59 | 2.98 | 0.83 | 70.26 | 0.68 | 1.55 | 10.48 | 3.10 |
|  |  | 6 | 1.23 | 56.27 | 1.08 | 1.57 | 12.57 | 2.71 | 1.08 | 57.40 | 1.07 | 1.57 | 12.50 | 2.82 | 1.01 | 57.57 | 1.06 | 1.56 | 12.37 | 3.04 |
|  |  | 9 | 1.52 | 44.25 | 3.11 | 1.59 | 14.69 | 2.69 | 1.33 | 45.14 | 3.09 | 1.58 | 14.62 | 2.85 | 1.24 | 45.27 | 3.06 | 1.58 | 14.47 | 2.85 |
|  | 0.35 | 0 | 0.75 | 81.24 | 0.52 | 1.60 | 9.42 | 6.29 | 0.65 | 82.86 | 0.52 | 1.60 | 9.37 | 7.47 | 0.60 | 83.11 | 0.51 | 1.59 | 9.27 | 8.36 |
|  |  | 3 | 1.34 | 58.31 | 0.88 | 1.57 | 13.04 | 2.20 | 1.17 | 59.47 | 0.88 | 1.56 | 12.98 | 2.38 | 1.09 | 59.65 | 0.87 | 1.56 | 12.84 | 2.50 |
|  |  | 6 | 1.67 | 41.62 | 3.05 | 1.58 | 15.42 | 2.22 | 1.46 | 42.46 | 3.04 | 1.58 | 15.34 | 2.43 | 1.36 | 42.59 | 3.01 | 1.57 | 15.19 | 2.55 |
|  |  | 9 | 2.18 | 34.99 | 5.76 | 1.62 | 18.70 | 2.17 | 1.89 | 35.69 | 5.73 | 1.62 | 18.61 | 2.35 | 1.77 | 35.80 | 5.67 | 1.62 | 18.42 | 2.45 |
| 0.12 | 0.15 | 0 | 0.53 | 74.99 | 0.64 | 1.64 | 8.15 | 5.46 | 0.46 | 76.48 | 0.63 | 1.64 | 8.11 | 6.43 | 0.43 | 76.72 | 0.63 | 1.63 | 8.03 | 8.06 |
|  |  | 3 | 1.23 | 58.15 | 2.30 | 1.56 | 15.21 | 1.33 | 1.07 | 59.30 | 2.29 | 1.56 | 15.14 | 1.66 | 1.00 | 59.49 | 2.27 | 1.55 | 14.98 | 1.99 |
|  |  | 6 | 1.36 | 40.58 | 4.80 | 1.63 | 18.74 | 1.36 | 1.19 | 41.39 | 4.78 | 1.62 | 18.65 | 1.53 | 1.10 | 41.51 | 4.72 | 1.62 | 18.46 | 1.72 |
|  |  | 9 | 1.61 | 28.60 | 8.27 | 2.27 | 23.08 | 1.51 | 1.40 | 29.17 | 8.22 | 2.27 | 22.97 | 1.56 | 1.31 | 29.26 | 8.15 | 2.26 | 22.73 | 1.75 |
|  | 0.25 | 0 | 0.60 | 79.93 | 0.80 | 2.47 | 9.81 | 5.17 | 0.52 | 81.52 | 0.80 | 2.47 | 9.75 | 5.86 | 0.49 | 81.77 | 0.79 | 2.46 | 9.65 | 7.52 |
|  |  | 3 | 1.38 | 55.32 | 5.76 | 1.64 | 21.58 | 1.47 | 1.21 | 56.42 | 5.73 | 1.64 | 21.47 | 1.60 | 1.12 | 56.58 | 5.67 | 1.63 | 21.25 | 1.68 |
|  |  | 6 | 1.57 | 31.83 | 6.90 | 2.22 | 25.06 | 1.34 | 1.37 | 32.46 | 6.86 | 2.22 | 24.94 | 1.51 | 1.28 | 32.56 | 6.80 | 2.21 | 24.68 | 1.58 |
|  |  | 9 | 1.79 | 26.45 | 8.77 | 2.46 | 31.74 | 1.34 | 1.56 | 26.98 | 8.72 | 2.46 | 31.58 | 1.60 | 1.46 | 27.07 | 8.64 | 2.45 | 31.25 | 1.64 |
|  | 0.35 | 0 | 0.78 | 74.17 | 0.81 | 2.64 | 9.84 | 4.25 | 0.67 | 75.65 | 0.81 | 2.64 | 9.79 | 4.81 | 0.63 | 75.89 | 0.80 | 2.63 | 9.68 | 6.03 |
|  |  | 3 | 1.64 | 41.77 | 5.27 | 1.66 | 30.29 | 1.19 | 1.43 | 42.61 | 5.25 | 1.65 | 30.13 | 1.40 | 1.33 | 42.73 | 5.19 | 1.65 | 29.83 | 1.48 |
|  |  | 6 | 1.50 | 19.36 | 6.70 | 2.01 | 30.62 | 1.06 | 1.31 | 19.74 | 6.66 | 2.01 | 30.47 | 1.17 | 1.22 | 19.80 | 6.60 | 2.00 | 30.15 | 1.38 |
|  |  | 9 | 1.92 | 14.03 | 9.43 | 2.26 | 37.18 | 1.09 | 1.68 | 14.31 | 9.37 | 2.26 | 36.99 | 1.12 | 1.57 | 14.35 | 9.29 | 2.25 | 36.61 | 1.28 |
| 0.2 | 0.15 | 0 | 0.62 | 53.33 | 0.94 | 2.41 | 8.77 | 3.12 | 0.52 | 54.40 | 0.93 | 2.40 | 8.73 | 3.44 | 0.49 | 54.56 | 0.92 | 2.40 | 8.64 | 4.30 |
|  |  | 3 | 1.95 | 30.13 | 5.36 | 1.60 | 32.34 | 0.61 | 1.69 | 30.74 | 5.33 | 1.60 | 32.17 | 0.69 | 1.58 | 30.83 | 5.27 | 1.60 | 31.84 | 0.83 |
|  |  | 6 | 2.17 | 16.50 | 6.95 | 2.17 | 36.55 | 0.57 | 1.89 | 16.82 | 6.90 | 2.17 | 36.36 | 0.60 | 1.77 | 16.87 | 6.84 | 2.16 | 35.98 | 0.72 |
|  |  | 9 | 2.69 | 10.85 | 10.27 | 2.29 | 38.71 | 0.55 | 2.34 | 11.06 | 10.21 | 2.29 | 38.51 | 0.52 | 2.20 | 11.09 | 10.11 | 2.28 | 38.11 | 0.66 |
|  | 0.25 | 0 | 0.76 | 39.92 | 1.15 | 2.46 | 9.54 | 2.99 | 0.66 | 40.71 | 1.15 | 2.45 | 9.48 | 3.26 | 0.62 | 40.84 | 1.13 | 2.44 | 9.38 | 3.93 |
|  |  | 3 | 2.16 | 24.81 | 8.71 | 1.66 | 40.35 | 0.65 | 1.89 | 25.31 | 8.66 | 1.65 | 40.15 | 0.66 | 1.76 | 25.39 | 8.58 | 1.65 | 39.73 | 0.69 |
|  |  | 6 | 2.42 | 10.07 | 10.17 | 2.24 | 42.31 | 0.57 | 2.11 | 10.27 | 10.11 | 2.23 | 42.10 | 0.65 | 1.98 | 10.30 | 10.02 | 2.22 | 41.66 | 0.65 |
|  |  | 9 | 2.94 | 0.97 | 13.71 | 2.25 | 47.57 | 0.49 | 2.56 | 0.99 | 13.63 | 2.25 | 47.33 | 0.53 | 2.40 | 1.00 | 13.50 | 2.24 | 46.84 | 0.59 |
|  | 0.35 | 0 | 0.79 | 30.82 | 0.57 | 2.40 | 9.40 | 2.92 | 0.68 | 31.43 | 0.57 | 2.40 | 9.36 | 3.17 | 0.65 | 31.53 | 0.56 | 2.38 | 9.25 | 3.69 |
|  |  | 3 | 2.78 | 3.68 | 9.44 | 1.76 | 45.70 | 0.51 | 2.54 | 3.75 | 9.38 | 1.75 | 45.47 | 0.58 | 1.52 | 3.76 | 9.29 | 1.75 | 44.99 | 0.71 |
|  |  | 6 | 4.21 | 1.94 | 11.63 | 2.27 | 52.43 | 0.61 | 3.00 | 1.98 | 11.57 | 2.27 | 52.18 | 0.52 | 1.95 | 1.99 | 11.46 | 2.26 | 51.63 | 0.61 |
|  |  | 9 | 4.80 | 0.00 | 18.27 | 2.32 | 53.83 | 0.42 | 4.18 | 0.56 | 17.26 | 2.31 | 53.55 | 0.48 | 3.91 | 0.64 | 15.27 | 2.30 | 53.00 | 0.55 |
